# TDP-43 Mutation Affects Stress Granule Dynamics in Differentiated NSC-34 Motoneuron-Like Cells

**DOI:** 10.3389/fcell.2021.611601

**Published:** 2021-06-08

**Authors:** Qiao Ding, Justin Chaplin, Matthew J. Morris, Massimo A. Hilliard, Ernst Wolvetang, Dominic C. H. Ng, Peter G. Noakes

**Affiliations:** ^1^Faculty of Medicine, School of Biomedical Sciences, The University of Queensland, Brisbane, QLD, Australia; ^2^Clem Jones Centre for Ageing Dementia Research, Queensland Brain Institute, The University of Queensland, Brisbane, QLD, Australia; ^3^Queensland Brain Institute, The University of Queensland, Brisbane, QLD, Australia; ^4^Australian Institute for Bioengineering and Nanotechnology, The University of Queensland, Brisbane, QLD, Australia

**Keywords:** TDP-43, stress granules, translation, motor neurons, cytoplasmic inclusions, amyotrophic lateral sclerosis

## Abstract

Amyotrophic Lateral Sclerosis (ALS) is characterized by degeneration of motor neurons in the brain and spinal cord. Cytoplasmic inclusions of TDP-43 are frequently reported in motor neurons of ALS patients. TDP-43 has also been shown to associate with stress granules (SGs), a complex of proteins and mRNAs formed in response to stress stimuli that temporarily sequester mRNA translation. The effect of pathogenic TDP-43 mutations within glycine-rich regions (where the majority of ALS-causing TDP-43 mutations occur) on SG dynamics in motor neurons is poorly understood. To address this issue, we generated murine NSC-34 cell lines that stably over-express wild type TDP-43 (TDP-43^*WT*^) or mutant forms (ALS-causing TDP-43 mutations TDP-43^*A*315T^ or TDP-43^*M*337V^). We then differentiated these NSC-34 lines into motoneuron-like cells and evaluated SG formation and disassembly kinetics in response to oxidative or osmotic stress treatment. Wild type and mutant TDP-43 appeared to be largely retained in the nucleus following exposure to arsenite-induced oxidative stress. Upon arsenite removal, mutant TDP-43 clearly accumulated within HuR positive SGs in the cytoplasm, whereas TDP-43^*WT*^ remained mostly within the nucleus. 24 h following arsenite removal, all SGs were disassembled in both wild type and mutant TDP-43 expressing cells. By contrast, we observed significant differences in the dynamics of mutant TDP-43 association with SGs in response to hyperosmotic stress. Specifically, in response to sorbitol treatment, TDP-43^*WT*^ remained in the nucleus, whereas mutant TDP-43 relocalized to HuR positive SGs in the cytoplasm following exposure to sorbitol stress, resulting in a significant increase in TDP-43 SG numbers. These SGs remained assembled for 24 h following removal of sorbitol. Our data reveal that under certain stress conditions the rates of SG formation and disassembly is modulated by TDP-43 mutations associated with ALS, and suggest that this may be an early event in the seeding of insoluble cytoplasmic inclusions observed in ALS.

## Introduction

Amyotrophic lateral sclerosis (ALS) is the most common adult-onset motor neuron disease. About 90% of patients exhibit sporadic ALS (sALS), whereas in ∼10% of familial ALS patients (fALS) a genetic cause has been identified, including mutations in the TDP-43 gene (trans-active response (TAR)-DNA binding protein 43 kDa) (Nowicka et al., 2019). Both sporadic and familial ALS patients present with TDP-43-containing insoluble cytoplasmic inclusions (Arai et al., 2006; Neumann et al., 2006), suggesting that TDP-43 plays a central role in ALS pathogenesis. This pathological accumulation of TDP-43 has also been seen in cortical neurons in a subset of ALS patients that present with frontotemporal lobar degeneration (FTLD) (Berning and Walker, 2019; Laferriere et al., 2019). How TDP-43-related dysfunction results in motor neuron degeneration and ALS remains largely unresolved (Birsa et al., 2019; Hergesheimer et al., 2019; Prasad et al., 2019).

TDP-43 is predominantly localized to the nucleus where it mediates a variety of RNA processing functions (Lagier-Tourenne et al., 2010; Weskamp and Barmada, 2018), but can also translocate to the cytoplasm where it is thought to play a role in mRNA transport (Alami et al., 2014). In response to stress, TDP-43 can be recruited into stress granules (SGs), a complex between mRNAs and proteins such as TIA-1, G3BP or HuR (Colombrita et al., 2009; Liu-Yesucevitz et al., 2010; Dewey et al., 2011; McDonald et al., 2011), that is thought to play a role in the transient stalling of mRNA translation (Protter and Parker, 2016). Whilst the formation of SGs is reversible, it is suspected that repeated cellular stress over time may lead to a transition to irreversible SGs assembly, a process associated with pathological aggregation of SG components, including TDP-43 (Kedersha and Anderson, 2002; Dewey et al., 2012; Khalfallah et al., 2018; Ivanov et al., 2019). Here we investigate whether ALS-causing mutations in TDP-43 affect the kinetics of SG formation or disassembly in motoneuron-like cells derived from the murine NSC-34 cell line, a neuroblastoma-spinal cord hybrid cell line developed by Cashman et al. (1992).

## Materials and Methods

### Plasmid Construction

The following TDP-43 expression constructs were made: wild-type human TDP-43 fused in frame to enhanced green fluorescent protein (hTDP-43^*WT*–*EGFP*^, referred to as TDP-43^*WT*^), mutated hTDP-43 with methionine changed to valine at codon 337–fused to EGFP (hTDP-43^*M*337V–*EGFP*^), and mutated hTDP-43 with alanine changed to threonine at codon 315 fused to EGFP (hTDP-43^*A*315T–*EGFP*^), referred to as TDP-43^*A*315T^ or TDP-43^*M*337V^ thereafter. These mutations were generated by PCR amplification from a plasmid bearing TDP-43^*WT*^ under the control of the *C. elegans Punc-25* promoter. TDP-43^*WT*^-containing plasmid was used to make the two TDP-43 mutations M337V and A315T. The TDP-43 sequences (WT and mutated) within the *Punc-25* plasmids/vectors were amplified by PCR using the forward primer with XhoI restriction site, and the reverse primer with BamHI restriction site from the *Punc-25* plasmid (Supplementary Table 1). The PCR products were then digested with XhoI and BamHI and inserted into pEGFP-C1 plasmid backbone. All the three plasmids then contained TDP-43 fused in frame to EGFP (TDP-43^*WT*^, TDP-43^*A*315T^, and TDP-43^*M*337V^), as verified by Sanger sequencing.

### Generation and Validation of Stable NSC-34 Cells Expressing TDP-43

NSC-34 cells (Cashman et al., 1992), were purchased from CELLutions Biosystem INC. (Catalog # CLU140). The cells were seeded at 60,000/well in a 24-well plate. The next day cells were transfected with Lipofectamine 2000 (Thermo Fisher Scientific, Catalog # 11668027). Each expression plasmid contained a Neomycin selection cassette, allowing for drug selection of single clones. This selection was achieved using Geneticin (Gibco, Catalog # 10131-035), at a concentration of 500 μg/ml. Each transfected well was treated with Geneticin for ∼3–4 weeks. Clonal colonies were picked and seeded onto 48-well plates and expanded–only clones with green fluorescent cells were expanded. For flow cytometric analyses, NSC-34 cells were trypsinized with Trypsin-EDTA (Gibco, Catalog # 25200056) and suspended in phosphate buffered saline pH 7.4 (PBS) and analyzed by BD LSRFortessa^TM^ X-20 flow cytometer. This procedure allowed us to select TDP-43 clones of different genotype expressing similar levels of EGFP fluorescence.

### Western Blotting

**FIGURE 1:**
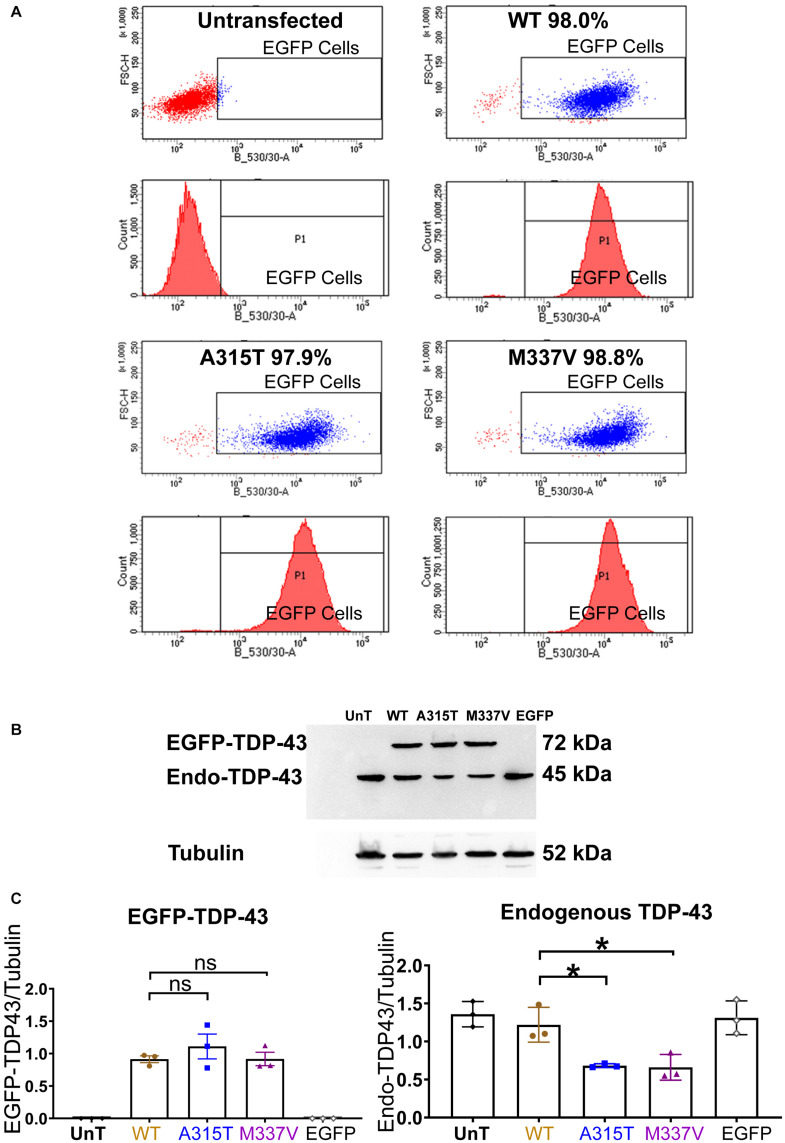
Stable expression of TDP-43 in NSC-34 cell lines. **(A)** Shows a series of fluorescent flow cytometry analyses of NSC-34 cell clones that have been transfected with EGFP-TDP-43 expression plasmids, compared to untransfected NSC-34 cells (top left). In WT (top right), A315T (bottom left) and M337V (bottom right), the red peaks within the EGFP expression gate indicates that more than 95% of the cells from these 3 TDP-43 clones express EGFP. For WT, 98.0% of cells are EGFP positive; for A315T, 97.9% of cells are EGFP positive; and for M337V, 98.8% of cells are EGFP positive. The top left graph represents non-transfected NSC-34 cells that serve as a negative control (i.e., all cells are EGFP negative and thus lie to the left of the EGFP selection gate). Note these plots are representatives of two experiments with different passage numbers of transfected NSC-34 cell clones, showing similar results. **(B)** A western blot that reveals similar expression for WT, A315T, M337V exogenous TDP-43 protein. The lower band in each lane is the expression of endogenous NSC-34 TDP-43 protein. The first and last lanes are controls. The first lane (UnT) refers to non-transfected NSC-34 cells, which have only endogenous TDP-43 expression; the last lane refers to NSC-34 cells transfected with EGFP-only plasmid, again containing only endogenous TDP-43 expression. **(C)** Left graph shows the means and SEMs of the immuno-stained band densities for exogenous TDP-43 WT (brown), A315T (Blue) and M337V (purple) proteins. **(C)** Right graph shows the levels of endogenous NSC-34 TDP-43 expression; within the TDP-43 WT clone, similar levels of exogenous and endogenous TDP-43 protein were observed. In A315T and M337V the level of endogenous NSC-34 TDP-43 dropped by about 50%. Tubulin was used as a loading control. *n* = 3, One-way ANOVA, *P* = 0.0171 for TDP-43^*WT*^ VS TDP-43^*A*315T^, *P* = 0.0142 for TDP-43^*WT*^ VS TDP-43^*M*337V^. *p < 0.05.

Western blot was used to validate that TDP-43 clones selected by EGFP-based flow cytometry did indeed express similar levels of transfected TDP-43 (see Figure 1). In addition, Western blot was also used to examine the location of TDP-43 both endogenous and exogenous. For whole cell lysates, cells were rinsed once with PBS and then lysed in 1× SDS loading buffer followed by boiling for 5 min. For nuclear and cytoplasmic preparations, cells were washed (PBS) and lysed in a Cytoplasmic Lysis Buffer (10 mM HEPES pH 7.9, 1.5 mM MgCl_2_, 0.5 mM 1,4-dithiothreitol, 0.05% (v/v), NP-40, protease inhibitor cocktail tablet), incubated on ice (10 min), and centrifuged at 4°C at 1,000 × g for 10 min. The supernatant was collected as a cytoplasmic extract. The pellet was resuspended in Nuclear Lysis Buffer (50 mM Tris-HCl pH 7.5, 0.5 mM EDTA, 100 mM NaCl, 1% Triton X-100, 1% (w/v) Sodium Deoxycholate, 1 mM Sodium Fluoride, 2 mM Sodium Orthovanadate, 10% Glycerol, 1 mM PMSF, protease inhibitor cocktail tablets), incubated on ice for 15 min and centrifuged at 4°C at 16,000 × g for 10 min. The resulting supernatant was collected as a crude nuclear extract.

Protein extracts were loaded and electrophoresed on 12% SDS-PAGE gels and then transferred onto nitrocellulose membranes. The membranes were blocked with 5% non-fat milk in PBS with Tween 20 (PBST) at room temperature for 1 h, and incubated with rabbit anti-TDP-43 (Cell Signaling Technology, Catalog #3448S, 1:1,000), mouse anti α-tubulin (Cell Signaling Technology, Catalog #3873S, 1:2,000), JNK1/2 (BD Pharmigen, Catalog #554285, 1:1,000), phosphor (pT183/pY185)-JNK1/2 (BD Pharmigen, Catalog #612541), GAPDH (Abcam, Catalog #ab9485, 1:5,000) or Lamin A/C (Cell Signaling Technology, Catalog #4777, 1:1,000) at 4°C overnight. After 3 washes with PBST, the membranes were incubated with anti-rabbit or anti-mouse HRP-conjugated secondary IgG (Jackson ImmunoResearch, West Baltimore, PA, Code: 115-035-003 and 111-035-003, 1:30,000 dilute in PBST) at room temperature for 1 h, followed by 3 washes in PBST. The proteins were visualized by WESTAR NOVA 2.0 (CYANAGEN, Bologna, Catalog # XLS071). The image density of immuno-stained bands normalized to anti α-tubulin (loading control) was assessed using a Kodak image station 4000 MM (Carestream Health, Rochester, NY) and Image J software (Schindelin et al., 2012). Whole blot images appear in Supplementary Data.

### Stress Stimulation in Stably Transfected Differentiated NSC-34 Cells

NSC-34 cells were seeded at 1,200/cm^2^ on round coverslips and maintained in growth medium containing Dulbecco’s modified Eagles Medium (DMEM) supplemented with 10% Fetal Calf Serum (FCS). After 24 h, when the cells were approximately 20% confluent, the culture medium was replaced with differentiation medium, consisting of DMEM/F12 supplemented with 0.5% FCS, 1% MEM Non-Essential Amino Acid (Gibco, Catalog # 11140050) and 2 μM *all-trans* retinoic acid (Sigma, Catalog # R2625). After 5 days of differentiation, the cells were treated for 30 min with either sodium arsenite (Sigma, Catalog # S7400) at 0.5 mM or D-sorbitol (Sigma, Catalog # S3889) at 0.5 M, as per similar studies (Colombrita et al., 2009; Liu-Yesucevitz et al., 2010; Dewey et al., 2011). Untreated cells were used as the negative control. Stress stimulated cells were either fixed and stained for SG formation or allowed to recover in differentiation medium by removing the stressor for between 1 and 24 h, before being fixed and stained (detailed below). The experiments were repeated 3 times each on different days (i.e., 3 independent experiments, *n* = 3).

### Immunofluorescence

After sorbitol or sodium arsenite treatment for 30 min or recovering from stress for between 1 and 24 h, the cells were rinsed twice with PBS, and fixed with 4% paraformaldehyde (PFA) in PBS for 10 min at room temperature. After rinsing 3 times in PBS, cells were treated with blocking buffer containing 2% bovine serum albumin (BSA) and 0.1% Triton X-100 for 30 min at room temperature. Cells were next incubated overnight at 4°C with primary antibodies HuR (Santa Cruz Biotechnology, Catalog # sc-5261, 1:300), TDP-43 (Cell Signaling Technology, Catalog # 3448S, 1:200), G3BP (Abcam, Catalog # ab 56574, 1:300) and GFP (Abcam, Catalog # ab5450, 1:2,000) diluted in blocking buffer. Cells were washed 3 times with PBS for 5 min prior to incubation at room temperature with the appropriate Alexa-conjugated second antibodies for 1 h: Alexa Fluor^®^ 488 donkey anti-goat (Invitrogen, Catalog # A32814), Alexa Fluor^®^ 555 goat anti-mouse (Invitrogen, Catalog # A32727) each diluted at 1:500 in blocking buffer. The coverslips containing the immuno-stained NSC-34 cells were mounted with Prolong Diamond anti-fade medium containing DAPI (Invitrogen, Catalog # P36962), and placed on glass slides. Images were captured using a 63X Glycerol NA 1.3 objective mounted onto a Leica DMi8 SP8 Inverted Confocal microscope. Immuno-staining procedures and image exposure times were standardized between genotype for a given set of experiments. Resolution was set up at 1,024 × 1,024 DPI for bi-directional scanning. Pinhole size was set at 1. The gain per channel across all experiments was the same. Finally, image panels were assembled in Imaris software for analysis. Surface was created based on the cytoplasmic red signals for quantification of granule number and size. There were no changes to gamma settings, curves or input levels. The images were exported as TIFF files in Imaris. All TIFF image files were then imported into Adobe Photoshop (Adobe Inc., United States) where only the brightness and contrast were uniformly adjusted across all panels. These adjustments did not obscure or remove any digital information present in the original raw images. Finally, all images and graphs were assembled and labeled in Adobe Illustrator (Abode Inc., United States) and converted into PDF figure files.

### Analysis and Statistical Comparisons

The images were analyzed with Imaris software. To quantify SGs number and size, surface was created by the cytoplasmic red signals for granule number and size quantification. In Imaris the “*create surface*” tool was used, under “*contour*,” drawing mode “*circle*” was chosen, and the radium of 0.1 μm was set up for all the images to ensure the smallest SGs were included. As all the images were taken under the same settings, the brightness was not adjusted when analyzing. All the SGs were selected and surface was created. SGs number and size were automatically created. The number of SGs was indicated by total number of Surfaces. SG size was divided into three groups: less than 0.3 μm^2^ (≤0.3 μm^2^), greater than 0.5 μm^2^ (≥0.5 μm^2^), and between 0.3 and 0.5 μm^2^. 23–30 cells were analyzed per individual experiments. GraphPad Prism 8 software was employed to undertake all statistical analyses and to create the presented graphs. Multiple group analyses with single independent variables were performed by using one-way ANOVAs. To compare within groups, Dunnett’s multiple comparisons was used. *P* < 0.05 was considered as statistically significant. The Pearson’s correlation coefficient analyses were performed in Image J by using Colocalization Finder. The nuclei were removed and only the cytoplasmic granules were analyzed. The image channel was split and type was changed to 8-bit. Using “Colocalization Finder” plugin, the Pearson’s correlation was shown as “Pearson’s Rr.”

## Results

### Wild Type and Mutant Human TDP-43 Are Localized to the Nucleus in NSC-34 Motoneuron-Like Cells

Under basal physiological conditions, TDP-43 is predominantly localized to the nucleus (Winton et al., 2008; Fallini et al., 2012). In response to cellular stress, TDP-43 has been reported to translocate to the cytoplasm and co-localize with SGs (Colombrita et al., 2009; Liu-Yesucevitz et al., 2010; Dewey et al., 2011; Meyerowitz et al., 2011). In order to determine if ALS-causing mutations of TDP-43 enhance the association with SGs in motoneuron-like cells, we first stably transfected NSC-34 cells with TDP-43^*WT*^, fALS-causing mutations TDP-43^*A*315T^, and TDP-43^*M*337V^, that were N-terminally tagged with Enhanced Green Fluorescent Protein (EGFP), or transfected with EGFP alone. Following antibiotic selection, single cell-derived colonies were picked, expanded, and assessed for EGFP expression by FACS. Cell lines with equivalent levels of EGFP expression (Figure 1A) were subjected to western blotting revealing equivalent levels of exogenous TDP-43 expression in each of the lines (Figures 1B,C, left graph). Interestingly, this resulted in a concomitant downregulation of endogenous TDP-43 protein expression in the lines that expressed the TDP-43^*A*315T^ or TDP-43^*M*337V^ mutant proteins, but not in the NSC-34 line that expressed TDP-43^*WT*^ (Figures 1B,C). These observations suggest that compared to wild type TDP-43, mutated TDP-43 has a more dramatic effect in decreasing the level of endogenous TDP-43 (Xu et al., 2011).

**FIGURE 2:**
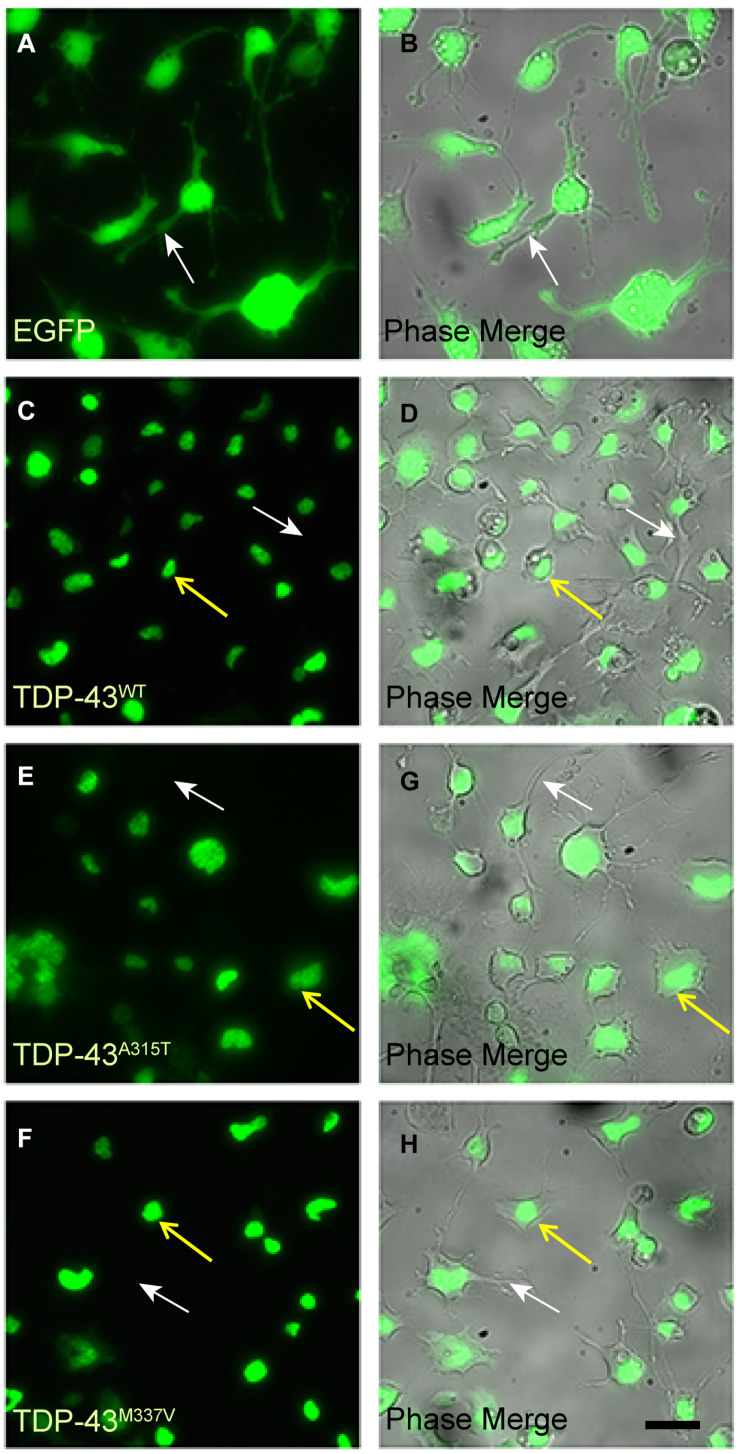
EGFP-TDP-43 is confined to the nucleus of TDP-43^*WT*^, TDP-43^*A*315T^, and TDP-43^*M*337V^ stably transfected differentiated NSC-34 cells. **(A,B)** EGFP fluorescent and corresponding phase images of control NSC-34 cells transfected with EGFP only. EGFP is present within the nuclei, cytoplasm and neurites (white arrows) of the cells. By contrast, EGFP fluorescence in TDP-43^*WT*^ **(C,D)**, TDP-43^*A*315T^ **(E,G)**, and TDP-43^*M*337V^ **(F,H)** is confined to the nuclei (yellow arrows), and not present in cytoplasm of neurites (white arrows). Scale bar = 20 μm for all panels.

We next differentiated the WT and mutant EGFP-TDP-43 lines and the EGFP-only NSC-34 line into motoneuron-like cells and examined the subcellular localization of EGFP (Figure 2). We observed that EGFP fluorescence in motoneuron-like cells stably transfected with TDP-43^*WT*^, TDP-43^*A*315T^ and TDP-43^*M*337V^ appeared localized to the nuclei (yellow arrows in Figures 2C–H). As expected, NSC-34 cells stably expressing EGFP (control expression plasmid) exhibited EGFP fluorescence in both the nucleus and the cytoplasm (Figures 2A,B). Thus, under basal conditions both WT and mutant TDP-43 proteins were predominantly localized to the nuclei in NSC-34 derived motoneuron-like cells. We also examined the localization of endogenous TDP-43 in NSC-34 cells stably expressing either TDP-43^*WT*^ or EGFP-only under both oxidative and osmotic stress conditions and verified that both exogenously expressed and endogenous TDP-43 (visualized by double immunostaining for GFP and TDP-43) were largely retained in the nucleus prior to and following stress treatment (Supplementary Figures 2A, 3A). Moreover, we performed nuclear-cytoplasmic fractionation of NSC-34 stable cells and confirmed that exogenously expressed EGFP-tagged TDP-43^*WT*^ and endogenous TDP-43 (delineated by a difference in molecular weight from EGFP fusion) was predominantly retained in nuclear fractions. We did, however, note low amounts of exogenous and endogenous TDP-43 were present within cytoplasmic fractions following exposure to stress treatment (Supplementary Figure 4).

We also investigated whether endogenous or exogenously expressed TDP-43 was truncated in our cell lines, given the reported accumulation of C-terminal truncated TDP-43 fragments associated with ALS pathology, and that overexpression of TDP-43 can result in the production of C-terminal fragments (Li et al., 2015). Immunoblots of WT and mutant NSC34 cells untreated and stress treated probed with anti-TDP43 did not reveal the presence of truncated TDP-43 protein (Supplementary Figures 4, 5A,B). However, given the limited sensitivity of immunoblots, we cannot rule out the presence of TDP-43 C-terminal fragments in the cytoplasm in stress treated NSC-34 cells. Nevertheless, these immunoblots did reveal that the levels of endogenous TDP-43 and EGFP-tagged mutant and wild-type TDP-43 did not change with stress treatment in our NSC-34 cell lines. Taken together, our studies confirm that the subcellular localization and expression levels of full-length EGFP-tagged TDP-43 were similar to, and reflective of endogenous TDP-43 in our stable NSC-34 cell lines under basal and stress conditions.

### SGs Dynamics of Wild Type and Mutant TDP-43 NSC-34 Cells Subjected to Oxidative Stress

**FIGURE 3:**
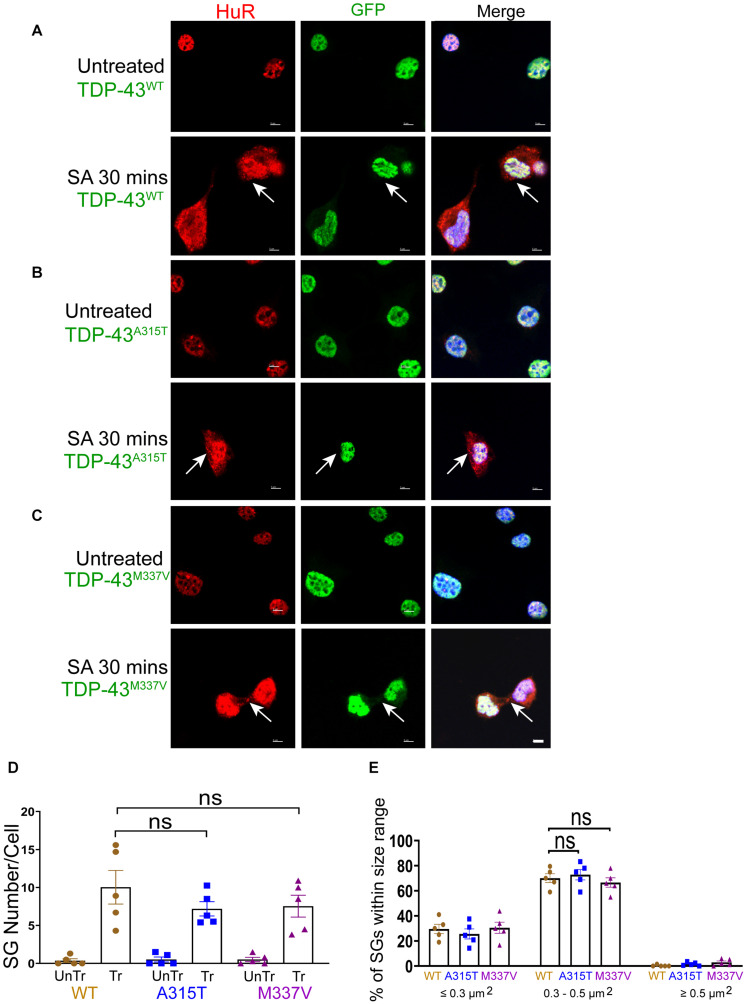
WT and mutant TDP-43 remain confined to nuclei upon oxidative stress, with no difference in SGs number and size. **(A)** NSC-34 cells that express TDP-43^*WT*^. Top panels, untreated NSC-34 cells expressing HuR in the nuclei (red), and with TDP-43^*WT*^ also present in the nuclei (green). Bottom panels, these cells were treated with sodium arsenite (SA) at 0.5 mM for 30 min, and Hur positive SGs (arrows) were formed in the cytoplasm, whereas TDP-43^*WT*^ appeared to remain in the nuclei. **(B)** NSC-34 cells that express TDP-43^*A*315T^. Top panels, untreated NSC-34 cells expressing HuR in the nuclei (red), and TDP-43^*A*315T^ present in the nuclei (green). Bottom panel, these cells were treated with SA at 0.5 mM for 30 min, and SGs (arrows) formed in the cytoplasm, but no TDP-43^*A*315T^ granules were found. **(C)** NSC-34 cells that express TDP-43^*M*337V^. Top panels, untreated NSC-34 cells express HuR in the nuclei (red), and TDP-43^*M*337V^ also present in the nuclei (green). Bottom panels, these cells were treated with SA at 0.5 mM for 30 min, and SGs (arrows) formed in the cytoplasm, but no TDP-43^*M*337V^granules were found. Scale bar = 5 μm. **(D)** Mean number of Hur positive SGs per cell in NSC-34 cells stably expressing the following: TDP-43^*WT*^ (WT, brown); TDP-43^*A*315T^ (A315T, blue); and TDP-43^*M*337V^ (M337V, purple), compared with untreated cells (UnTr) for each phenotype. There was no significant difference across 3 genotypes. *n* = 5, One-way ANOVA, WT VS A315T *P* = 0.4176, WT VS M337V *P* = 0.5084. The number of cells quantified were between 115 and 150. **(E)** Shows size histograms of Hur positive SGs per genotype which were divided as ≤0.3 μm^2^, 0.3–0.5 μm^2^, and ≥0.5 μm^2^. Most SGs were 0.3–0.5 μm^2^ and there were no differences between genotypes. *n* = 5, One-way ANOVA, WT VS A315T ns *P* = 0.3036, WT VS M337V ns *P* = 0.8026.

**FIGURE 4:**
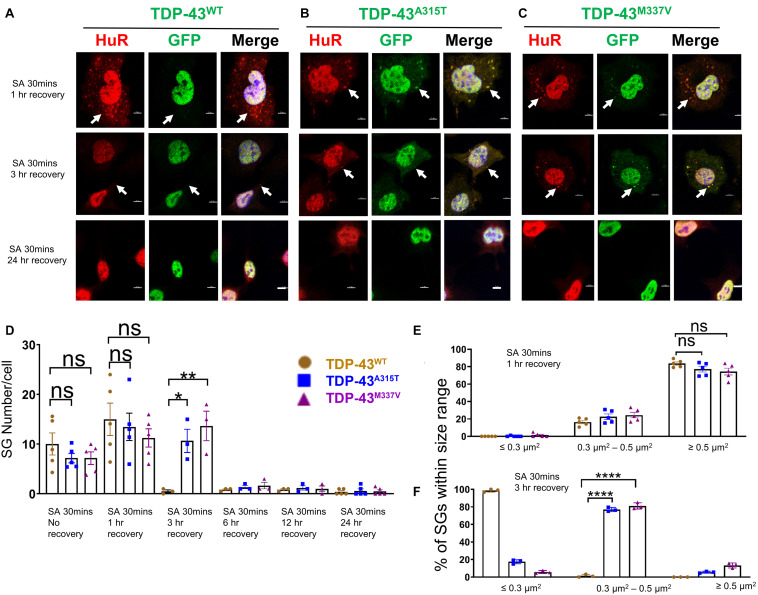
Mutant TDP-43 translocates to cytoplasm and co-localizes with SGs upon removal of oxidative stress. **(A)** NSC-34 cells that express TDP-43^*WT*^ were treated with SA at 0.5 mM for 30 min and then SA was removed allowing the cells to recover for 1 h (top panel), 3 h (middle panel) or 24 h (bottom panel). Hur positive SGs clearly formed (arrows in top panel), but no TDP-43 granules were found (middle in top panel). Middle panel shows the Hur positive SGs were almost disassembled (arrows showed the very few SGs left) at 3 h recovery. Bottom panel shows no Hur positive SGs at 24 h recovery. **(B)** NSC-34 cells that express TDP-43^*A*315T^ were treated with SA at 0.5 mM for 30 min and then SA was removed allowing the cells to recover for 1 h (top panel), 3 h (middle panel) or 24 h (bottom panel). Hur positive SGs were visible, and they co-localized with TDP-43^*A*315T^ (arrows in top panel) after oxidative stressor was removed for 1 h; Pearson’s correlation coefficient was 0.91. Hur positive SGs were still visible, and they co-localized with TDP-43^*A*315T^ (arrows in middle panel) after oxidative stressor was removed for 3 h; Pearson’s correlation coefficient was 0.92. Both disappeared after the stressor was removed for 24 h (bottom panel). **(C)** NSC-34 cells that express TDP-43^*M*337V^ were treated with SA at 0.5 mM for 30 min and then SA was removed allowing the cells to recover for 1 h (top panel), 3 h (middle panel) or 24 h (bottom panel). Hur positive SGs were visible, and they colocalized with TDP-43^*M*337V^ (arrows in middle panel) after oxidative stressor was removed for 1 h; Pearson’s correlation coefficient was 0.85. Hur positive SGs were still visible, and they co-localized with TDP-43^*M*337V^ (arrows in middle panel) after oxidative stressor was removed for 3 h; Pearson’s correlation coefficient was 0.87. Both disappeared after stressor was removed for 24 h (bottom panel). Scale bar = 5 μm. **(D)** Mean number of Hur positive SGs per cell in NSC-34 cells stably expressing WT, A315T, and M337V after SA treatment for 30 min (No Recovery), SA treatment for 30 min and 1 h recovery, SA treatment for 30 min and 3 h recovery, SA treatment for 30 min and 12 h recovery, and SA treatment for 30 min and 24 h recovery. There was no significant difference in SGs number in NSC-34 cells expressing wild type TDP-43 or mutated TDP-43 (A315T and M337V) following oxidative stress for 30 min, or 30 min treatment and 1 h recovery. *n* = 5, One-way ANOVA. For SA 30 min 1 h recover, WT VS A315T *P* = 0.8882, WT VS M337V *P* = 0.5243. The number of cells quantified were between 115 and 150. There was a significant difference in SG number in NSC-34 cells expressing wild type TDP-43 or mutant TDP-43 following oxidative stress for 30 min treatment and 3 h recovery. *n* = 3, One-way ANOVA, WT VS A315T *P* = 0.0292, WT VS M337V *P* = 0.0095. **(E)** Shows size histograms of Hur positive SGs per genotype after SA treatment for 30 min and removal of SA for 1 h. There were no differences between genotypes. *n* = 5, One-way ANOVA, WT VS A315T ns *P* = 0.5333, WT VS M337V ns *P* = 0.4742. **(F)** Shows size histograms of Hur positive SGs per genotype after SA treatment for 30 min and removal of SA for 3 h. There was significant difference between genotypes. *n* = 3, One-way ANOVA, WT VS A315T *P* < 0.0001, WT VS M337V *P* < 0.0001. *p ≤ 0.05; **p ≤ 0.01; ***p ≤ 0.001; ****p ≤ 0.0001.

We next sought to determine stress-stimulated effects on exogenously expressed mutant TDP-43 compared to the wild-type protein. When we exposed TDP-43^*WT*^, TDP-43^*A*315T^, and TDP-43^*M*337V^ expressing NSC-34 motoneuron-like cells to sodium arsenite for 30 min, we observed the emergence of HuR positive SGs but, as indicated above, did not detect translocation of TDP-43 into the cytoplasm (Figures 3A–C). We observed similar results when utilizing G3BP as an SG marker (Supplementary Figure 6). However, 1 h following removal of sodium arsenite TDP-43^*A*315T^ and TDP-43^*M*337V^, but not TDP-43^*WT*^, translocated to the cytoplasm and co-localized with HuR positive SGs (compare arrows in top panels of Figure 4A with arrows in top panels of 4B,C) and G3BP positive SGs (Supplementary Figure 7), suggesting a delayed association of mutated TDP-43 with SGs following sodium arsenite-induced stress. The Pearson’s correlation coefficient for TDP-43^*A*315T^ and TDP-43^*M*337V^ expressing NSC-34 motoneuron-like cells was 0.91 and 0.85, respectively, indicating a near complete overlap between TDP-43 and HuR fluorescence signals.

Next, we wanted to determine if EGFP was cleaved from our EGFP-fused TDP-43-protein, which would indicate the presence of TDP-43-C terminal fragments. To assess this possibility, we performed immunostaining with antibodies to TDP-43 and GFP and showed that the TDP-43 staining did overlap with GFP staining (Supplementary Figure 2B, merge), suggesting that EGFP-TDP-43 was not cleaved (see also immuno-blots **S4**, and **S5**). Interestingly, 3 h following arsenite removal, SGs in TDP-43^*WT*^ expressing cells were almost disassembled (Figure 4A, middle panel), whereas SGs in mutant TDP-43 expressing cells remained in the cytoplasm (Figures 4B,C middle panel), suggesting delayed disassembly of mutant TDP-43 granules. The Pearson’s correlation coefficient of A315T for 3 h recovery post SA 30 min treatment was 0.92, and that of M337V was 0.87, indicating that TDP-43 remained in HuR positive SGs. 24 h following arsenite removal, SGs across all three 3 genotypes were completely disassembled and no EGFP signal was detected in the cytoplasm of either WT or mutated TDP-43 expressing cells (Figures 4A–C bottom panels and graph in D; Supplementary Figure 8).

Despite previous reports suggesting that the glycine-rich region of TDP-43, where the A315T and M337V are located (Da Cruz and Cleveland, 2011), determines the association of TDP-43 with SGs (Dewey et al., 2011), we did not detect any differences in SGs number (Figures 3D, 4D) at SA 30 min treatment, SA 30 min treatment plus 1 h recovery across the genotypes; however, there were more SGs remaining in mutant TDP-43 expressing cells at SA 30 min treatment plus 3 h recovery. We also observed a shift in SGs size from medium (0.3–0.5 μm^2^) at 30 min SA treatment to large (≥0.5 μm^2^) size post SA treatment (1 h) across all genotypes (Figures 3E, 4E). Interestingly, the large SGs (≥0.5 μm^2^) post SA treatment (1 h) became medium sized (3 h) (Figures 4E,F), suggesting a dynamic change of assembly and disassembly of SGs. Together these findings indicate that in our model system TDP-43 mutations do not in themselves result in altered SGs number or kinetics of formation when forming SGs, but TDP-43 does become associated with SGs following the removal of SA, and the presence of mutant TDP-43 delayed the clearance of SGs after oxidative stress was removed.

### Mutant but Not Wild Type TDP-43 Associates With Cytoplasmic SGs in Response to Hyperosmotic Stress

**FIGURE 5:**
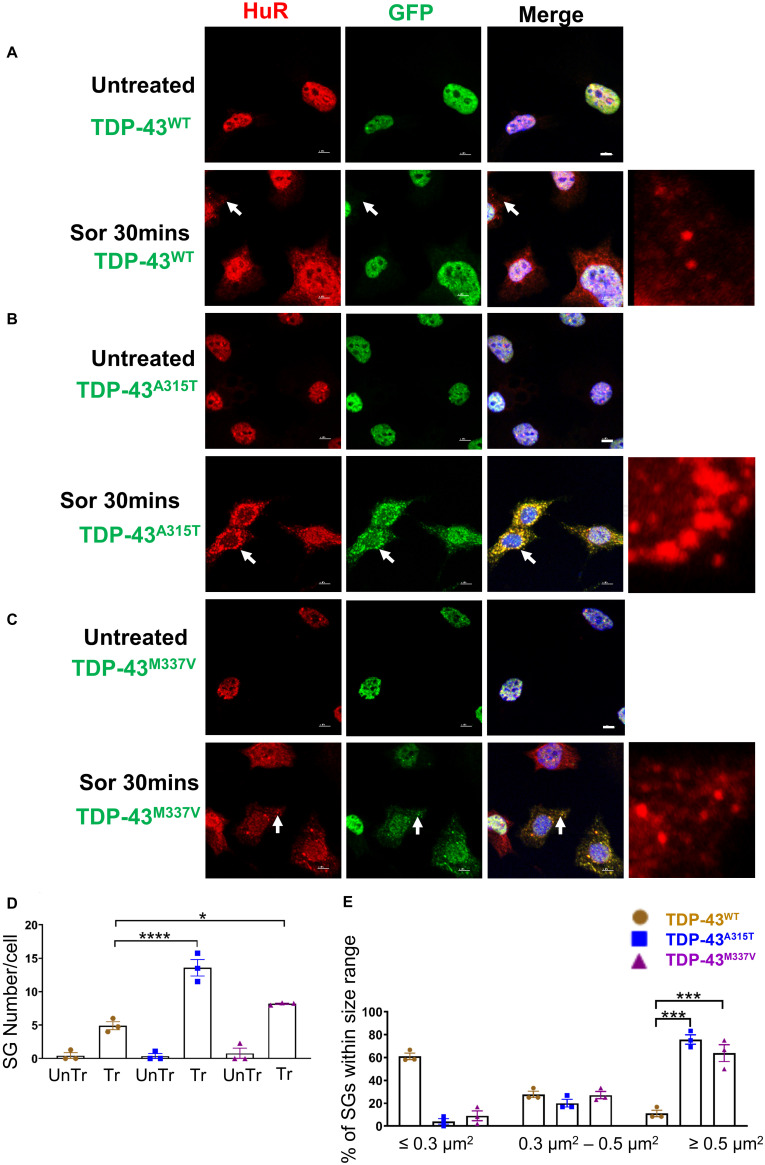
Mutant TDP-43 causes increase in SGs number and size and colocalized with HuR positive SGs in the cytoplasm in response to osmotic stress. NSC-34 cells treated for 30 min with 0.5 M sorbitol compared to untreated NSC-34 cells in the following conditions: **(A)** cells expressing TDP-43^*WT*^; **(B)** cells expressing TDP-43^*A*315T^; and **(C)** Cells expressing TDP-43^*M*337V^. **(A)** Formation of HuR positive stress granules in response to sorbitol treatment. TDP-43^*WT*^ did not appear to translocate to these SGs, but remained within the nucleus. Right side picture is a zoomed image of the area indicated by the arrows. By contrast, **(B,C)** show that sorbitol-induced Hur positive SGs were also positive for TDP-43—indicating that mutated TDP-43 did translocate into the cytoplasm to associate with SGs. Arrows indicate the position of HuR positive SGs with respect to TDP-43. Scale bar = 5 μm. Right side picture is a zoomed image of the area indicated by the arrow. At Sor treatment for 30 min, the Pearson’s correlation coefficient for A315T was 0.97, and for M337V was 0.96. **(D)** Shows the mean number of Hur positive SGs per cell in NSC-34 cells stably expressing TDP-43^*WT*^ (brown), TDP-43^*A*315T^ (blue), and TDP-43^*M*337V^ (purple). There were significantly more SGs in NSC-34 cells expressing mutated TDP-43 (A315T and M337V) compared to WT following osmotic stress. *n* = 3, One-way ANOVA, WT VS A315T **** *P* = 0.0001, WT VS M337V *P = 0.0378. The number of cells quantified were between 69 and 90. **(E)** Shows size histograms of Hur positive SGs per genotype. In TDP-43 mutant NSC-34 cells, 75.8 and 63.9% of Hur positive SGs in TDP-43^*A*315T^ and TDP-43^*M*337V^ expressing cells, respectively, were >0.5 μm^2^, compared with only 11.1% SGs in TDP-43^*WT*^ expressing NSC-34 cells. WT VS A315T ***P = 0.0002, WT VS M337V ***P = 0.0006.

Because the dynamics of SG assembly/disassembly can differ in response to different stress stimuli (Kedersha et al., 2008; Vanderweyde et al., 2013; Chen and Liu, 2017), and sorbitol-induced osmotic stress was previously shown to direct TDP-43 to SGs in human embryonic kidney 293 cells (HEK293T) and primary cultured glia cells (Dewey et al., 2011), we next subjected NSC-34 motoneuron-like cells to sorbitol treatment (Finan and Guilak, 2010; Miermont et al., 2013; Chandler-Brown et al., 2015; Gonzalez et al., 2016). Our data showed that following 30-min treatment with sorbitol effectively induced HuR-positive SGs in each of the TDP-43 expressing NSC-34 motoneuron-like cells, but did not result in translocation of TDP-43^*WT*^ to SGs (arrows in Figure 5A, lower row compared to upper row). By contrast, this same treatment resulted in robust translocation of TDP-43^*A*315T^ and TDP-43^*M*337V^ to SGs, as indicated by the co-localization with HuR immunofluorescence (arrows in Figure 5B, lower row, and higher view panel far right, arrows in Figure 5C, lower row and high view panel far right). The Pearson’s correlation coefficient for TDP-43^*A*315T^ and TDP-43^*M*337V^ expressing NSC-34 motoneuron-like cells was 0.97 and 0.96, respectively, indicating a near complete overlap between TDP-43 and HuR fluorescence signals.

**FIGURE 6:**
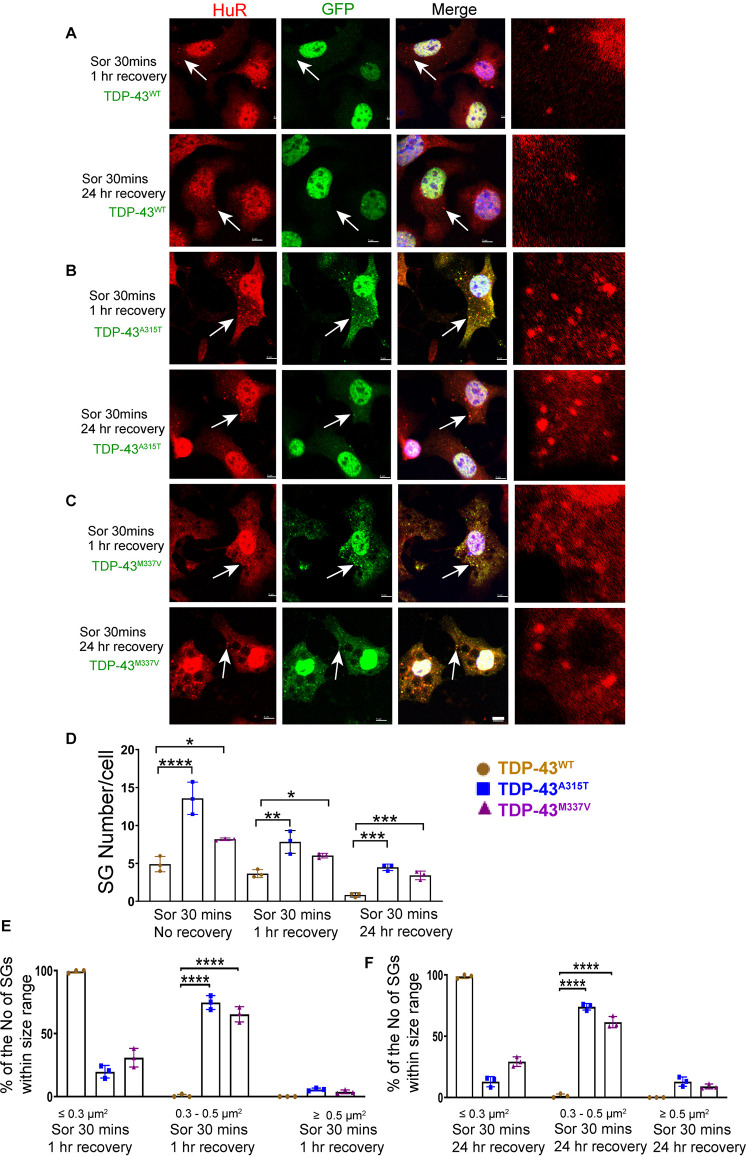
Mutant TDP-43 remains associated with more and larger SGs after osmotic stressor was removed, post 1 and 24 h. Shown are TDP-43 transfected NSC-34 cells that had been treated for 30 min with 0.5M sorbitol, then imaged at 1 and 24 h post its removal for the following set of NSC-34 cells: **(A)** cells expressing TDP-43^*WT*^; **(B)** cells expressing TDP-43^*A*315T^; and **(C)** cells expressing TDP-43^*M*337V^. In **(A)**, arrow indicates the locations of Hur positive SGs—these SGs did appear to co-express TDP-43, and by 24 h most Hur positive SGs had disassembled. In **(B)**, at 1 h post stress, arrows indicate colocalization of SGs and TDP-43^*A*315T^. By 24 h there were persistent Hur positive SGs present. In **(C)**, similar to **(B)**, SGs were seen at 1 and 24 h post removal of sorbitol (arrows). Scale bar = 5 μm. Right side pictures in **(A–C)** are the zoomed area for the arrow indicated area. At sorbitol treatment for 30 min and 1 h recovery, the Pearson’s correlation coefficient for A315T was 0.88, and for M337V was 0.84. At sorbitol treatment for 30 min and 24 h recovery, the Pearson’s correlation coefficient for A315T was 0.75, and for M337V was 0.89. **(D)** Shows the mean number Hur positive SGs per cell in NSC-34 cells stably expressing the following: WT (brown), A315T (blue) and M337V (purple) at 3 time points: sorbitol 30 min, sorbitol 30 min and removal for 1 h, sorbitol 30 min and removal for 24 h. There were significantly more Hur positive SGs in NSC-34 cells expressing mutated TDP-43 (A315T and M337V) compared to TDP-43^*WT*^ at sorbitol 30 min treatment with no recovery, at sorbitol 30 min treatment with 1 h recovery, and at sorbitol 30 min treatment with 24 h recovery. *n* = 3, One-way ANOVA, For Sor 30 min no recovery, WT VS A315T ****P = 0.0001, WT VS M337V *P = 0.0378. For sorbitol (Sor) 30 min 1 h recovery, WT VS A315T **P = 0.0029, WT VS M337V *P = 0.0374. For Sor 30 min 24 h recovery, WT VS A315T ***P = 0.0001, WT VS M337V ***P = 0.0007. The number of cells quantified were between 69 and 90. **(E,F)** Show size histograms of Hur positive SGs per genotype which were divided as ≤ 0.3 μm^2^, 0.3–0.5 μm^2^ and ≥ 0.5 μm^2^. **(E)** Shows removal of osmotic stress for 1 h. In TDP-43 mutant NSC-34 cells, Hur positive SGs were larger with most (74.7% for A315T, 65.3% for M337V) within 0.3–0.5 μm^2^, compared to those in TDP-43^*WT*^ NSC-34 cells (99.3% were less than 0.3 μm^2^). *n* = 3, One-way ANOVA, WT VS A315T ****P = 0.0001, WT VS M337V ****P = 0.0001. **(F)** Shows removal of osmotic stress for 24 h. Hur positive SGs in mutant cells were consistently larger (74% for A315T, 61.5% for M337V were 0.3–0.5 μm^2^), whereas 98.7% were less than 0.3 μm^2^ in WT cells. *n* = 3, One-way ANOVA, WT VS A315T ****P = 0.0001, WT VS M337V ****P = 0.0001.

To ensure that our GFP staining was detecting exogenous EGFP-fused TDP43 and not cleavage of EGFP from TDP-43, which could indicate the presence of TDP-43 C-terminal fragments, we undertook immuno-staining with antibodies to TDP-43 and GFP. Our staining showed that the TDP-43 did overlap with GFP staining (Supplementary Figure 3B, merge), suggesting that EGFP-TDP43 was not cleaved following sorbitol treatment (see immuno-blots Supplementary Figures 4, 5). We did note that our anti-TDP43 immunostaining did reveal cytoplasmic TDP-43 that was not associated with SG (Supplementary Figure 3B). When we used G3BP as an SG marker, we found that upon sorbitol treatment, mutant TDP-43-EGFP overlapped with G3BP, but G3BP also appeared to localize independently of SGs under both stress conditions (Supplementary Figures 7, 9, 10). Our data further showed that the average number of HuR positive SGs per cell following sorbitol exposure (30 min) was the highest in NSC-34 cell expressing TDP-43^*A*315T^ (∼14 SGs per cell), followed by TDP-43^*M*337V^ (∼8 SGs per cell) and TDP-43^*WT*^ (∼5 SGs per cell; summarized in Figure 5D), compared to a minimal number of SGs present within in non-sorbitol treated cells for all TDP-43 genotypes (Figure 5D). Furthermore, SGs in sorbitol treated TDP-43^*A*315T^ or TDP-43^*M*337V^ expressing cells were larger than in TDP-43^*WT*^ expressing cells (75.8% greater than 0.5 μm^2^ for A315T, and 63.9% greater than 0.5 μm^2^ for M337V; Figure 5E, examples are shown in the high view image panels in Figures 5A–C). Interestingly, these large mutant TDP-43-containing SGs were the same size as those SGs observed at 1 h post sodium arsenite treatment (compare Figure 4E with Figure 5E). The increased size and number of HuR positive SGs in sorbitol-treated NSC-34 cells, led us to hypothesize that A315T and M337V mutations in TDP-43 may inhibit SG resolution. Indeed, following 1 or 24 h post removal of sorbitol, the mean number of HuR positive SGs per cell had dropped dramatically in NSC-34 cells expressing TDP-43^*WT*^ (Figure 6D), whereas both A315T- and M337V-expressing NSC-34 cells still possessed significant numbers of HuR positive SGs (lower rows in Figures 6A–C, and quantified in Figure 6D) and consistently remained co-localized with TDP-43 (arrows in Figures 6B,C). Our data showed that these persistent SGs-containing mutated TDP-43 were reduced in size from those large ones observed during the stress treatment, to medium size post treatment (compare Figure 5E with Figures 6E,F). In addition, the Pearson’s correlation coefficient for TDP-43^*A*315T^ and TDP-43^*M*337V^ expressing NSC-34 motoneuron-like cells at 1 h post sorbitol treatment was 0.88 and 0.84, respectively, and that for TDP-43^*A*315T^ and TDP-43^*M*337V^ expressing NSC-34 motoneuron-like cells at 24 h post sorbitol treatment was 0.75 and 0.89, respectively, indicating a slight drop of overlap between TDP-43 and HuR fluorescence signals during recovery period.

## Discussion

In the present study, we employed the motoneuron-like cell line NSC-34, which is commonly used as an *in vitro* motoneuron model, to investigate the role of TDP-43 in ALS pathogenesis. We generated stable cell lines in which the exogenous wild type (**WT**) TDP-43 expression level was similar to exogenous mutant TDP-43. This approach has allowed us to study the dynamics of mutated TDP-43 association with HuR and G3BP positive stress granule formation, and in particular, the transition to irreversible SGs; all of which is closer to being physiologically relevant in a differentiated motoneuron-like cell stably expressing low levels of exogenous TDP-43 across all genotypes. Further, our *in vitro* study provides for ease of tracking SGs compared to transgenic mice. We have shown that both WT and mutant TDP-43 were present in the nuclei, which was reminiscent of ALS patients who harbor TDP-43 mutations, but do not show symptoms until a later age (Mackenzie et al., 2007; Wegorzewska et al., 2009; Deng et al., 2010). Our data also showed that under osmotic stress conditions mutant TDP-43 may exacerbate cytoplasm retention and colocalization with SGs, whereas under other stress conditions, such as oxidation, the selected translocation of mutated TDP-43 and its association with SGs was delayed, but appeared to slow the resolution of SGs from medium to small size. These observations suggest that different environmental stressors employ different mechanisms to regulate SG dynamics, and that mutated TDP-43 can interfere with these processes. This interference may be important for ALS pathology, as it signifies a possible disturbance in the normal nucleo-cytoplasmic shuttling and function of TDP-43 (Kelley and Paschal, 2007; Patel and Chu, 2011). It may also suggest that environmental stress and SG composition play a critical role in forming pathological TDP-43 granules, since the TDP-43 mutation alone does not cause accumulation of cytoplasmic protein aggregates which are observed in ALS patients.

### Optimizing the Localization of Exogenous TDP-43 for *in vitro* Modeling of TDP-43 Function

The pathology/toxicity of TDP-43 leading to cell death is thought to occur when it translocates from the nucleus to the cytoplasm (Chen-Plotkin et al., 2010; Walker et al., 2015). In this study, we have shown that under basal conditions, both WT and mutant TDP-43 (A315T or M337V) were restricted to the nuclei in NSC-34 motoneuron-like cells. This is consistent with findings from several studies that have reported WT and mutant TDP-43 to be located in the nuclei in stably transfected non-neuronal cell lines, including HEK293T (kidney) cells and HeLa (cervical cancer) cells (Ling et al., 2010; Dewey et al., 2011). By contrast, the location of exogenous TDP-43 (wild type and mutated) in transiently transfected cells (neuronal and non-neuronal) revealed both nuclear and cytoplasmic expression of transfected TDP-43 (Barmada et al., 2010; Walker et al., 2013; Liu-Yesucevitz et al., 2014). This has allowed researchers to explore the short-term role(s) of cytoplasmic TDP-43 in SG formation (Colombrita et al., 2009; Liu-Yesucevitz et al., 2010; Dewey et al., 2011; McDonald et al., 2011). Controlling the expression of exogenous TDP-43 is difficult with transient transfection, moreover transient transfections only allow for short-term cellular assessments. Here we have been able to examine NSC-34 motoneuron-like cells that have been stably transfected with WT or mutated TDP-43, with each clone expressing similar levels of exogenous TDP-43 (TDP-43^*WT*^, TDP-43^*A*315T^, and TDP-43^*M*337V^). This has allowed us to make meaningful examinations and comparisons of SGs dynamics across our NSC-34 cells expressing WT or mutated exogenous TDP-43 over long periods of time in culture (i.e., from 1 to 24 h).

When we examined the expression of exogenous TDP-43, we found that endogenous TDP-43 expression dropped in the presence of exogenous mutant TDP-43 compared with exogenous wild type TDP-43. These observations are consistent with the findings by Xu et al. who reported that transgenic mice overexpressing mutant TDP-43 reduced the levels of endogenous mouse TDP-43 (Xu et al., 2011), supporting the notion that the auto-regulation of TDP-43 has somehow been perturbed by the expression of mutated TDP-43 (e.g., see Ayala et al., 2011; Bembich et al., 2014; Sugai et al., 2019; Suk and Rousseaux, 2020).

### Wild-Type Exogenous and Endodenous TDP-43 Fail to Translocate to SGs in Response to Stress

It is now recognized that TDP-43 is involved in SG pathways (Dewey et al., 2012). Most studies have focused on how endogenous TDP-43 is induced to move out of nuclei to be associated with SGs. However, these studies have generated mixed results, which appear to be dependent on cell type and stressor. For example, Colombrita et al. (2009) employed undifferentiated NSC-34 cells and showed that sodium arsenite treatment caused recruitment of TDP-43 to be associated with SGs in the cytoplasm, whereas others have shown that this was not the case for SH-SY5Y neuronal cells (Meyerowitz et al., 2011). Other studies comparing sodium arsenite and sorbitol treatments showed that osmotic stress directed TDP-43 to SGs in HEK293T cells, whereas sodium arsenite did not (Dewey et al., 2011). These inconsistent findings suggest that various cell types and different stressors drive the formation of SGs containing distinct components, some including TDP-43 and some not (Anderson and Kedersha, 2008, 2009; Colombrita et al., 2009; Dewey et al., 2011; Meyerowitz et al., 2011; Markmiller et al., 2018). In our study, stable expression of exogenous WT TDP-43 in differentiated NSC-34 cells mostly remained nuclear following sodium arsenite or sorbitol treatment. We did observe in our cytoplasmic blots low levels of cytoplasmic TDP-43 (exogenous and endogenous). The fact that we did not observe endogenous or exogenous WT TDP-43 translocating to the cytoplasm to associate with SGs under either stress may indicate that non-mutated TDP-43 forms rapidly reversible protein complexes with SG proteins (i.e., within 30 min time period of stress treatment (Ling et al., 2010; Austin et al., 2014). By contrast, mutant TDP-43 may lead to an increase in the residency time in the cytoplasm, because mutations may have impaired the reversible dynamic interactions with SG components/proteins (Suk and Rousseaux, 2020). In support of these ideas are observations in human cell lines expressing WT and mutated endogenous TDP-43, as well as stably transfected Hela cell lines expressing exogenous WT and mutated TDP-43 (Ling et al., 2010). These studies demonstrated that mutant TDP-43 forms longer-lived complexes with SG proteins that are less easily resolved compared to the WT TDP-43 (Ling et al., 2010).

An alternate explanation for why we did not observe WT-TDP-43 EGFP associating with SGs in the cytoplasm in response to stress, could be due to the design of the expression construct. For example, tagging with EGFP may in itself alter the protein interaction properties of TDP-43 and this may in turn affect its trafficking. We think this is unlikely for the following reasons: (1), we have demonstrated that WT-TDP-43-EGFP behaves the same as endogenous mouse TDP-43, namely most of it remains in the nucleus without stress, during stress, and immediately following removal of stress, and (2), the movements of mutant TDP-43 to SGs in the cytoplasm were not prevented despite employing the same expression construct and identical n-terminus fusion to EGFP. These mutant TDP-43-EGFP proteins are able to move in and out of the nucleus in response to stress, in a similar manner shown by previous researchers (Liu-Yesucevitz et al., 2010; Besnard-Guerin, 2020). A novel aspect of our study is that we have used differentiated NSC-34 cells, which stably express similar levels of WT and mutated TDP-43, and for WT TDP-43, it behaves the same as endogenous mouse TDP-43.

### Mutated TDP-43 (A315T and M337V) Translocates to the Cytoplasm and Becomes Associated With SGs in Response to Environmental Stress

How mutations in TDP-43 cause SG dynamic disruption is a key question in understanding the relationship between TDP-43 and SGs. Dewey et al. showed larger SG formation in TDP-43 G348C mutant expressing cells during osmotic stress treatment (Dewey et al., 2011). Based on these observations, Dewey et al. (2011) proposed that SGs might contribute to the formation of TDP-43 pathological aggregates (“the seed hypothesis”; Dewey et al., 2012). In support of this idea, we have also observed that more and larger SGs were formed in mutant TDP-43 (A315T or M337V) expressing cells exposed to osmotic stress. All these mutations are located in the glycine-rich domain of TDP-43, which binds proteins, including SG-related proteins G3BP and TIA-1 (Freibaum et al., 2010; McDonald et al., 2011), suggesting that the glycine-rich domain of TDP-43 can affect SG formation and stability (Freibaum et al., 2010; McDonald et al., 2011). When we subjected WT and mutant TDP-43 expressing cells to sodium arsenite treatment, we observed an initial delay in mutant TDP-43 accumulating in SGs; specifically, mutant TDP-43 did not co-localize with SGs at 30 min of sodium arsenite treatment, but became localized to large SGs 1 h post recovery. Interestingly, these large SGs were of similar size to those containing mutated TDP-43 during 30 min sorbitol treatment, the difference being that with sorbitol treatment it was only the mutant TDP-43-containing SGs that were large, whereas all SGs (TDP-43-containing and not) were large post 1 h sodium arsenite treatment. These findings may suggest that mutated TDP-43 takes longer to shuttle out of the nucleus into the cytoplasm to associate with SGs in response to oxidative stress. We did note a discrepancy between our immunostaining and our immunoblot of cytoplasmic/nuclear fractionations; namely immunoblots of cytoplasmic fractions did not detect elevations in cytoplasmic mutant TDP-43 revealed by immunofluorescence. Whilst the specific reason of this is unclear, the differences are likely due to detection limits of the underlying methods and that contents of stress granules may not be readily detectable by cell fractionation and immunoblotting. We also noted that post recovery from sodium arsenite, the rate of SG dissembley was much slower in NSC34 cells expressing mutated TDP-43 when compared to WT-TDP-43, indicating the presence of mutated TPD-43 has slowed SG disassembely.

In addition, we found subtle differences in the colocalization of mutant TDP-43 with SG markers that were dependent on the stress conditions investigated. Specifically, mutant TDP-43 was only partially colocated with G3BP in response to hyperosmotic stress compared to a close overlap with HuR under identical stress conditions. Interestingly, this difference was not observed in response to sodium arsenite. This indicates that different stressors selectively recruit proteins to SGs with different dynamics. SGs are comprised of diverse constituent proteins that include mRNA-binding proteins (HuR), proteins involved in mRNA metabolism (G3BP), translation-related factors and other signaling proteins unrelated to RNA metabolism (Kedersha and Anderson, 2007; Kedersha et al., 2008). Given the distinct functions of HuR and G3BP, it is not surprising that there are subtle differences in their response to different stressors. It has been proposed that oxidative stress (sodium arsenite) and osmotic stress (sorbitol) induce distinct molecular pathways to SG formation (Hans et al., 2020). It could be that in our study, the delayed accumulation of mutated TDP-43 to SGs in response to oxidative stress compared to osmotic stress has revealed such differences. If true, then it would support the notion that the trafficking of mutant TDP-43 is highly dependent on environmental conditions, the cells type (differentiated or proliferating), and method of transfection and stress conditions.

### SGs Dynamics Is Altered in NSC-34 Cells Expressing A315T or M337V Mutated TDP-43, in a Stress-Dependent Fashion

One characteristic feature of SGs is that they are transient structures, which are resolved upon the removal of stress (Kedersha and Anderson, 2002; Kedersha et al., 2008; Ivanov et al., 2019). The rate of SG resolution differs widely across different neural cell types, from 1 h for glial cells to approximately 5 h for neurons (Khalfallah et al., 2018). This delay to eliminate SGs from neurons could provide an environment for the seeding of SGs toward insoluble cytoplasmic inclusions (Dewey et al., 2012), which is a feature of ALS pathology (Arai et al., 2006; Neumann et al., 2006). In support of this notion, our results revealed that mutant TDP-43-associated SGs remain assembled for 24 h following removal of osmotic stress, compared to the rapid disassembly of SGs containing wild type TDP-43. Previous studies have demonstrated that TDP-43 ALS-linked mutations, which include the ones we have studied here, are more stable than WT TDP-43 having enhanced interactions with other RNA-binding proteins such as FUS (Ling et al., 2010), another ALS-associated protein also found within insoluble cytoplasmic inclusions (Baron et al., 2013; Sama et al., 2013; Lenzi et al., 2015). It is possible that our ALS-linked TDP-43 mutations, under osmotic stress, alter the nature of TDP-43 interaction with proteins such as FUS and SG proteins G3BP and TIA-1 (McDonald et al., 2011; Baron et al., 2013; Sama et al., 2013; Lenzi et al., 2015; Markmiller et al., 2018) to maintain the presence of mutated TDP-43-containing SGs long after removal of stress. This continued presence of SGs caused by the stable expression of ALS-linked mutant TDP-43 might indicate a possible mechanism for how mutations in TDP-43 cause TDP-43 proteinopathy.

By contrast, when the same mutated TDP-43-expressing cells were treated with sodium arsenite, SGs containing mutated TDP-43 were present 1 h post removal of sodium arsenite, but had disassembled by 24 h. This maintenance of SGs containing mutated TDP-43 upon removal of osmotic stress, but clearance of such SGs upon oxidative stress, suggests that various stressors may play different roles in TDP-43 SG dynamics. It might also suggest that oxidative (sodium arsenite) and osmotic (sorbitol) stressors trigger distinct signaling pathways in SG assembly and disassembly, with some pathways resulting in more stable protein interactions with mutated TDP-43, an idea recently proposed by Hans and colleagues (Hans et al., 2020); see also (Markmiller et al., 2018). For example, sorbitol inactivates 70-kDa ribosomal protein S6 kinase (p70S6K), whereas arsenite activates p70S6K (Patel et al., 2002), and arsenite increases eEF2 phosphorylation, but not sorbitol (Patel et al., 2002). Our findings highlight the need to explore these ideas in future research to investigate how TDP-43 protein aggregates form within the motor neurons of ALS patients.

While our results are most directly relevant to ALS with TDP-43 mutations, we believe that our findings may also be informative of cytoplasmic inclusions seeded by wild-type TDP-43. Our current study shows that when expressed at low levels, mutated TDP-43 is correctly localized in the nucleus under basal conditions similar to the wild-type protein (whether ectopically expressed or endogenous). So the mutations in of themselves do not automatically result in cytoplasmic mislocalization/aggregation. Rather, mutant TDP-43 responses to stress (cytoplasmic localization to SGs) and recovery differ with subtle but important differences depending on the environmental stimuli. We suggest that this may be due to a degree of “stress loading” on neurons with one interpretation being that patient-identified mutations may accelerate or enhance the pathological process that would otherwise take time and repeated stress insults to manifest as mislocalization, aggregation or cellular toxicity, all of which give rise to interesting avenues for future investigations.

## Data Availability Statement

The original contributions presented in the study are included in the article/Supplementary Material, further inquiries can be directed to the corresponding author/s.

## Author Contributions

PGN, DN, QD, EW, and MAH designed the study. PGN, QD, and DN wrote the original manuscript. PGN, DN, MAH, and EW edited the versions of the manuscript, contributed to the data analysis, and presentation. QD performed the experiments and analyses. JC and QD constructed the TDP-43 expression constructs under the supervision of MAH. EW supervised the clonal section and characterization of transfected TDP-43 NSC-34 cells. MJM assisted QD with Western blots. QD and PGN assembled the figures. All authors contributed to the article and approved the submitted version.

## Conflict of Interest

The authors declare that the research was conducted in the absence of any commercial or financial relationships that could be construed as a potential conflict of interest.
