# Supplementary material for: TDP-43 Mutation Affects Stress Granule Dynamics in Differentiated NSC-34 Motoneuron-Like Cells
Source: Front Cell Dev Biol. 2021 Jun 8;9:611601. doi: 10.3389/fcell.2021.611601 (PMC8217991; doi:10.3389/fcell.2021.611601)
Supplement: Supplementary file 1 [file Data_Sheet_1.PDF]

Supplementary Table 1

Name	Sequence
EGFP TDP-43 XhoI F	CTGAGTCTCGAGGAatgtctgaatatattcgggtaac
EGFP TDP-43 BamHI R	CTGAGTGGATCCctacattccccagccaga
