# Supplementary material for: TDP-43 Mutation Affects Stress Granule Dynamics in Differentiated NSC-34 Motoneuron-Like Cells
Source: Front Cell Dev Biol. 2021 Jun 8;9:611601. doi: 10.3389/fcell.2021.611601 (PMC8217991; doi:10.3389/fcell.2021.611601)
Supplement: Supplementary file 2 [file Data_Sheet_2.pdf]

Supplementary Material

Supplementary Figure S1. Complete blot of Figure 1 for TDP-43 with molecular weight marker. A shows TDP-43 blotting. Top band shows exogenous (transfected) TDP-43 (EGFP-TDP-43) band with 72 kDa (just above 70 kDa, but below 100 kDa) is expressed in TDP-43^{WT}, TDP-43^{A315T}, TDP-43^{M337V} expressing NSC-34 cells (indicated by WT, A315T, M337V), but not expressed in un-transfected line (UnT) or EGFP-expressing line (EGFP). Bottom band shows endogenous TDP-43 (Endo-TDP-43) in all transgenic lines and un-transfected line (UnT). **B** shows tubulin band with 52 kDa (just below 55 kDa).

Supplementary Figure S2a. TDP-43 remains confined to nuclei upon oxidative stress. A NSC-34 cells that express TDP-43^{WT}. Top panels, untreated NSC-34 cells expressing HuR in the nuclei (red), with TDP-43^{WT} (green) and TDP-43 (magenta) also present in the nuclei. Bottom panels, these cells were treated with sodium arsenite (SA) at 0.5 mM for 30 mins, and HuR positive SGs (arrows) were formed in the cytoplasm, whereas TDP-43^{WT} and endogenous TDP-43 remained in the nuclei. **B** NSC-34 cells that express EGFP. Top panels, untreated NSC-34 cells expressing HuR in the nuclei (red), and EGFP present in the nuclei and cytoplasm (green). Bottom panel, these cells were treated with SA at 0.5 mM for 30 mins, and HuR positive SGs (arrows) formed in the cytoplasm, but no TDP-43 granules (magenta) were found (arrows). Scale bar = 5 µm.

Supplementary Figure S2b. TDP-43 remains confined to nuclei upon oxidative stress. A NSC-34 cells that express TDP-43^{A315T}. Top panels, untreated NSC-34 cells expressing HuR in the nuclei (red), with TDP-43^{A315T} (green) and TDP-43 (magenta) also present in the nuclei. Bottom panels, these cells were treated with sodium arsenite (SA) at 0.5 mM for 30 mins, and HuR positive SGs (arrows) were formed in the cytoplasm, whereas TDP-43^{A315T} remained in the nuclei. **B** NSC-34 cells that express TDP-43^{M337V}. Top panels, untreated NSC-34 cells expressing HuR in the nuclei (red), with TDP-43^{M337V} (green) and TDP-43 (magenta) also present in the nuclei. Bottom panels, these cells were treated with sodium arsenite (SA) at 0.5 mM for 30 mins, and HuR positive SGs (arrows) were formed in the cytoplasm, whereas TDP-43^{M337V} remained in the nuclei. Scale bar = 5 µm.

Supplementary Figure S3a. TDP-43 remains confined to nuclei upon osmotic stress. A NSC-34 cells that express TDP-43^{WT}. Top panels, untreated NSC-34 cells expressing HuR in the nuclei (red), with TDP-43^{WT} (green) and TDP-43 (magenta) also present in the nuclei. Bottom panels, these cells were treated with sorbitol (Sor) at 0.5 M for 30 mins, and HuR positive SGs (arrows) were formed in the cytoplasm, whereas TDP-43^{WT} and TDP-43 remained in the nuclei. **B** NSC-34 cells that express EGFP. Top panels, untreated NSC-34 cells expressing HuR in the nuclei (red), and EGFP present in the nuclei and cytoplasm (green). Bottom panel, these cells were treated with Sor at 0.5 M for 30 mins, and HuR positive SGs (arrows) formed in the cytoplasm, but no TDP-43 granules (magenta) were found (arrows). Scale bar = 5 µm.

Supplementary Figure S3b. TDP-43 translocates to cytoplasm upon osmotic stress. A NSC-34 cells that express TDP-43^{A315T}. Top panels, untreated NSC-34 cells expressing HuR in the nuclei (red), with TDP-43^{A315T} (green) and TDP-43 (magenta) also present in the nuclei. Bottom panels, these cells were treated with sorbitol (Sor) at 0.5 M for 30 mins, and HuR positive SGs (arrows) were formed in the cytoplasm, which colocalized with TDP-43^{A315T} and TDP-43. **B** NSC-34 cells that express TDP-43^{M337V}. Top panels, untreated NSC-34 cells expressing HuR in the nuclei (red), with TDP-43^{M337V} (green) and TDP-43 (magenta) also present in the nuclei. Bottom panels, these cells were treated with sorbitol (Sor) at 0.5 M for 30 mins, and HuR positive SGs (arrows) were formed in the cytoplasm, which colocalized with TDP-43^{M337V} and TDP-43. Scale bar = 5 µm.

Supplementary Figure S4. Endogenous and exogenous TDP-43 remain confined to nuclei upon oxidative or osmotic stress. A NSC-34 cells that express EGFP, TDP-43^{WT} (WT), TDP-43^{A315T} (A315T) were treated with Sodium Arsenite (**SA**) at 0.5 mM for 30 mins. Cytoplasmic protein and nuclear proteins were collected for both untreated cells (indicated as "**-**"), and treated cells (indicated as "SA"). Both EGFP-TDP-43 (i.e. exogenous TDP-43) and Endo-TDP-43 (i.e. endogenous TDP-43) were found in the nuclei (top two panels - exposed and overexposed blots respectively). Nuclear protein was confirmed by Lamin A/C, and cytoplasmic protein was confirmed by GAPDH. Only treated cells express phosphorylated JNK1/2 (indicated as "pJNK 1/2"), whereas both untreated and treated cells express JNK1/2. **B** NSC-34 cells that express EGFP, TDP-43^{WT}, TDP-43^{A315T} were treated with Sorbitol (Sor) at 0.5 M for 30 mins. Cytoplasmic protein and nuclear proteins were collected for both untreated cells (indicated as "**-**") and treated cells (indicated as "Sor"). Both EGFP-TDP-43 (i.e. exogenous TDP-43) and Endo-TDP-43 (i.e. endogenous TDP-43) were found in the nuclei (top two panels - exposed and overexposed blots respectively. Nuclear protein was confirmed by Lamin A/C, and cytoplasmic protein was confirmed by GAPDH. Only treated cells express phosphorylated JNK1/2 (indicated as "pJNK 1/2"), whereas both untreated and treated cells express JNK1/2. Note the second TDP-43 immuno-blot is an over exposure of upper most blot in each panel set.

Supplementary Figure S5a. Full length endogenous and exogenous TDP-43 immuno-stained bands were present upon oxidative or osmotic stress. NSC-34 cells that express TDP-43^{WT}, TDP-43^{A315T}, and TDP-43^{M337V} were treated with sodium arsenite (top blot) or sorbitol (bottom blot) for 30 mins and were recovered for 1 hr or 24 hrs. Protein lysates were collected and separated by Western Blot.

In each blot, there were only two TDP-43 immuno-stained bands, one representing exogenous EGFP-TDP-43, and the second representing endogenous TDP-43 (Endo-TDP-43). Phosphorylated JNK1/2 was used to confirm that only treated cells can express phosphorylated JNK1/2 (pJNK1/2), whereas all the cell lysates can express JNK1/2.

Supplementary Figure S5b. Overexposure of the Western blots shown in Figure S5a.

Supplementary Figure S6. WT and mutant TDP-43 remain confined to nuclei upon oxidative stress. A NSC-34 cells that express TDP-43^{WT}. Top panels, untreated NSC-34 cells expressing G3BP in the cytoplasm (red), and with TDP-43^{WT} present in the nuclei (green). Bottom panels, these cells were treated with sodium arsenite (SA) at 0.5 mM for 30 mins, and G3BP positive SGs (arrows) were formed in the cytoplasm, whereas TDP-43^{WT} remained in the nuclei. **B** NSC-34 cells that express TDP-43^{A315T}. Top panels, untreated NSC-34 cells expressing G3BP in the cytoplasm (red), and TDP-43^{A315T} present in the nuclei (green). Bottom panel, these cells were treated with SA at 0.5 mM for 30 mins, and SGs (arrows) formed in the cytoplasm, but no TDP-43^{A315T} granules were found. **C** NSC-34 cells that express TDP-43^{M337V}. Top panels, untreated NSC-34 cells express G3BP in the cytoplasm (red), and TDP-43^{M337V} present in the nuclei (green). Bottom panels, these cells were treated with SA at 0.5 mM for 30 mins, and SGs (arrows) formed in the cytoplasm, but no TDP-43^{M337V}granules were found. Scale bar = 5 μm.

Supplementary Figure S7. Mutant TDP-43 translocates to cytoplasm and co-localizes with SGs upon removal of oxidative stress. A NSC-34 cells that express TDP-43^{WT} were treated with SA at 0.5 mM for 30 mins and then sodium arsenite (SA) was removed allowing the cells to recover for 1 hr (top panel), or 24 hrs (bottom panel). G3BP positive SGs clearly formed (arrows in top panel), but no TDP-43 granules were found (middle in top panel). Bottom panel shows no G3BP positive SGs at 24 hr recovery. **B** NSC-34 cells that express TDP-43^{A315T} were treated with SA at 0.5 mM for 30 mins and then SA was removed allowing the cells to recover for 1 hr (top panel) or 24 hrs (bottom panel). G3BP positive SGs were visible, and they co-localized with TDP-43^{A315T} after oxidative stressor was removed for 1 hr (arrows). Both disappeared after the stressor was removed for 24 hrs (bottom panel). **C** NSC-34 cells that express TDP-43^{M337V} were treated with SA at 0.5 mM for 30 mins and then SA was removed allowing the cells to recover for 1 hr (top panel) or 24 hrs (bottom panel). G3BP positive SGs were visible, and they colocalized with TDP-43^{M337V} after oxidative stressor was removed for 1 hr (arrows). Both disappeared after stressor was removed for 24 hrs (bottom panel). Scale bar = 5 μm.

Supplementary Figure S8. WT and mutant TDP-43 are confined to nuclei upon removal of oxidative stress for 6 hrs and 12 hrs. A NSC-34 cells that express TDP-43^{WT} were treated with SA at 0.5 mM for 30 mins and then sodium arsenite (SA) was removed allowing the cells to recover for 6 hrs (top panel) and 12 hrs (bottom panel). There were no HuR positive SGs or TDP-43 positive granules. Both HuR (red) and TDP-43 (green) were expressed in the nuclei. Scale bar = 5 μm. **B** NSC-34 cells that express TDP-43^{A315T} were treated with SA at 0.5 mM for 30 mins and then SA was removed allowing the cells to recover for 6 hrs (top panel) and 12 hrs (bottom panel). There were no HuR positive SGs or TDP-43 positive granules. Both HuR (red) and TDP-43 (green) are expressed in

the nuclei. Scale bar = 5 µm. **C** NSC-34 cells that express TDP-43^{M337V} were treated with SA at 0.5 mM for 30 mins and then SA was removed allowing the cells to recover for 6 hrs (top panel) and 12 hrs (bottom panel). There were no HuR positive SGs or TDP-43 positive granules. Both HuR (red) and TDP-43 (green) are expressed in the nuclei. Scale bar = 5 µm.

Supplemental Figure S9. Mutant TDP-43 translocates to cytoplasm, and associates with G3BP positive SGs upon osmotic stress. A NSC-34 cells that express TDP-43^{WT}. Top panels, untreated NSC-34 cells expressing G3BP in the cytoplasm (red), and with TDP-43^{WT} present in the nuclei (green). Bottom panels, these cells were treated with sorbitol (Sor) at 0.5 M for 30 mins, and no G3BP positive SGs were formed in the cytoplasm, and TDP-43^{WT} remained in the nuclei. **B** NSC-34 cells that express TDP-43^{A315T}. Top panels, untreated NSC-34 cells expressing G3BP in the cytoplasm (red), and TDP-43^{A315T} present in the nuclei (green). Bottom panel, these cells were treated with Sor at 0.5 M for 30 mins, G3BP positive SGs and TDP-43^{A315T} granules were formed in the cytoplasm (arrows). **C** NSC-34 cells that express TDP-43^{M337V}. Top panels, untreated NSC-34 cells express G3BP in the cytoplasm (red), and TDP-43^{M337V} present in the nuclei (green). Bottom panels, these cells were treated with Sor at 0.5 M for 30 mins, G3BP-TDP-43^{M337V} positive SGs were formed in the cytoplasm (arrows). Scale bar = 5 µm.

Supplemental Figure S10. Mutant TDP-43 translocates to cytoplasm, and associates with G3BP positive SGs upon removal of osmotic stress. A NSC-34 cells that express TDP-43^{WT} were treated with sorbitol (Sor) at 0.5 M for 30 mins and then Sor was removed allowing the cells to recover for 1 hr (top panel), or 24 hrs (bottom panel). There were no G3BP positive SGs formed, and no TDP-43 granules were found either (middle in top panel). Bottom panel shows no G3BP positive SGs at 24 hr recovery. **B** NSC-34 cells that express TDP-43^{A315T} were treated with Sor at 0.5 M for 30 mins and then Sor was removed allowing the cells to recover for 1 hr (top panel) or 24 hrs (bottom panel). There were G3BP and TDP-43 positive SGs visible (arrows) after osmotic stressor was removed for 1 hr. By 24 hrs post removal of Sor, TDP-43 positive granules were still present (arrows in bottom panel). **C** NSC-34 cells that express TDP-43^{M337V} were treated with Sor at 0.5 M for 30 mins and then Sor was removed allowing the cells to recover for 1 hr (top panel) or 24 hrs (bottom panel). There were G3BP- TDP-43 positive SGs visible (arrows) after osmotic stressor was removed for 1 hr. These granules were also present 24 hrs after the stressor was removed (arrows in bottom panel). Scale bar = 5 µm.
