# Supplementary material for: TDP-43 Mutation Affects Stress Granule Dynamics in Differentiated NSC-34 Motoneuron-Like Cells
Source: Front Cell Dev Biol. 2021 Jun 8;9:611601. doi: 10.3389/fcell.2021.611601 (PMC8217991; doi:10.3389/fcell.2021.611601)
Supplement: Supplementary file 3 [file Data_Sheet_3.PDF]

S1

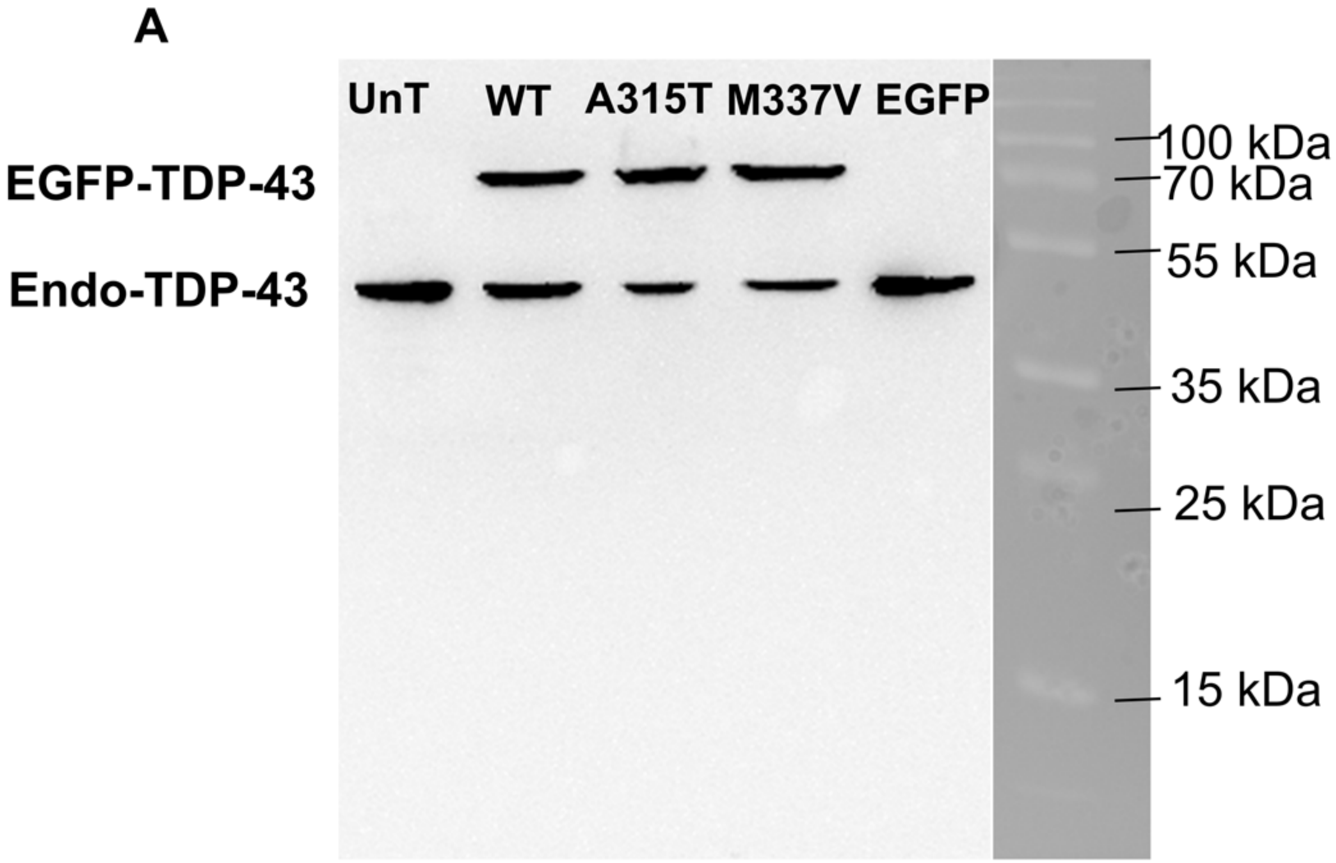
A
UnT
WT
A315T
M337V
EGFP
EGFP-TDP-43
Endo-TDP-43
100 kDa
70 kDa
55 kDa
35 kDa
25 kDa
15 kDa

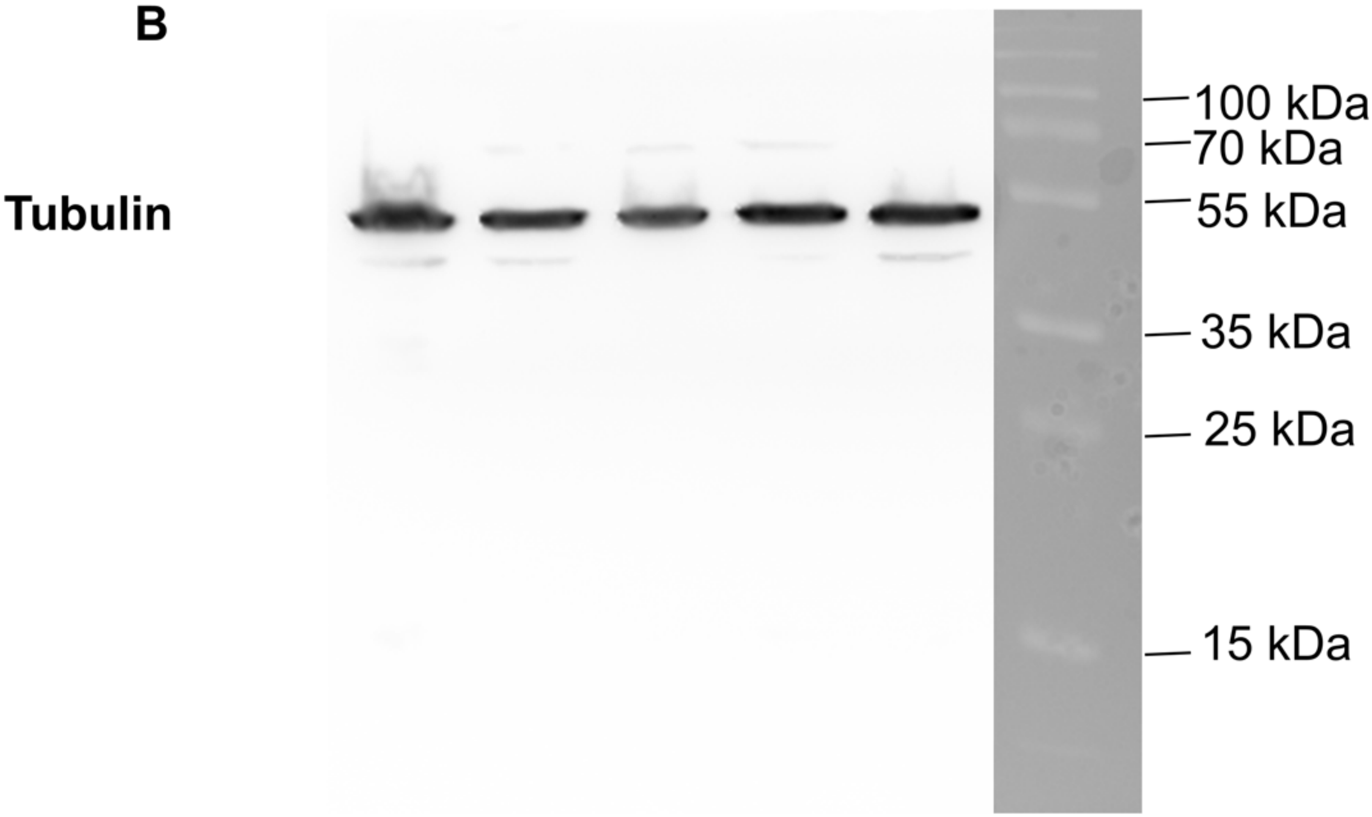
B
Tubulin
100 kDa
70 kDa
55 kDa
35 kDa
25 kDa
15 kDa

S2a

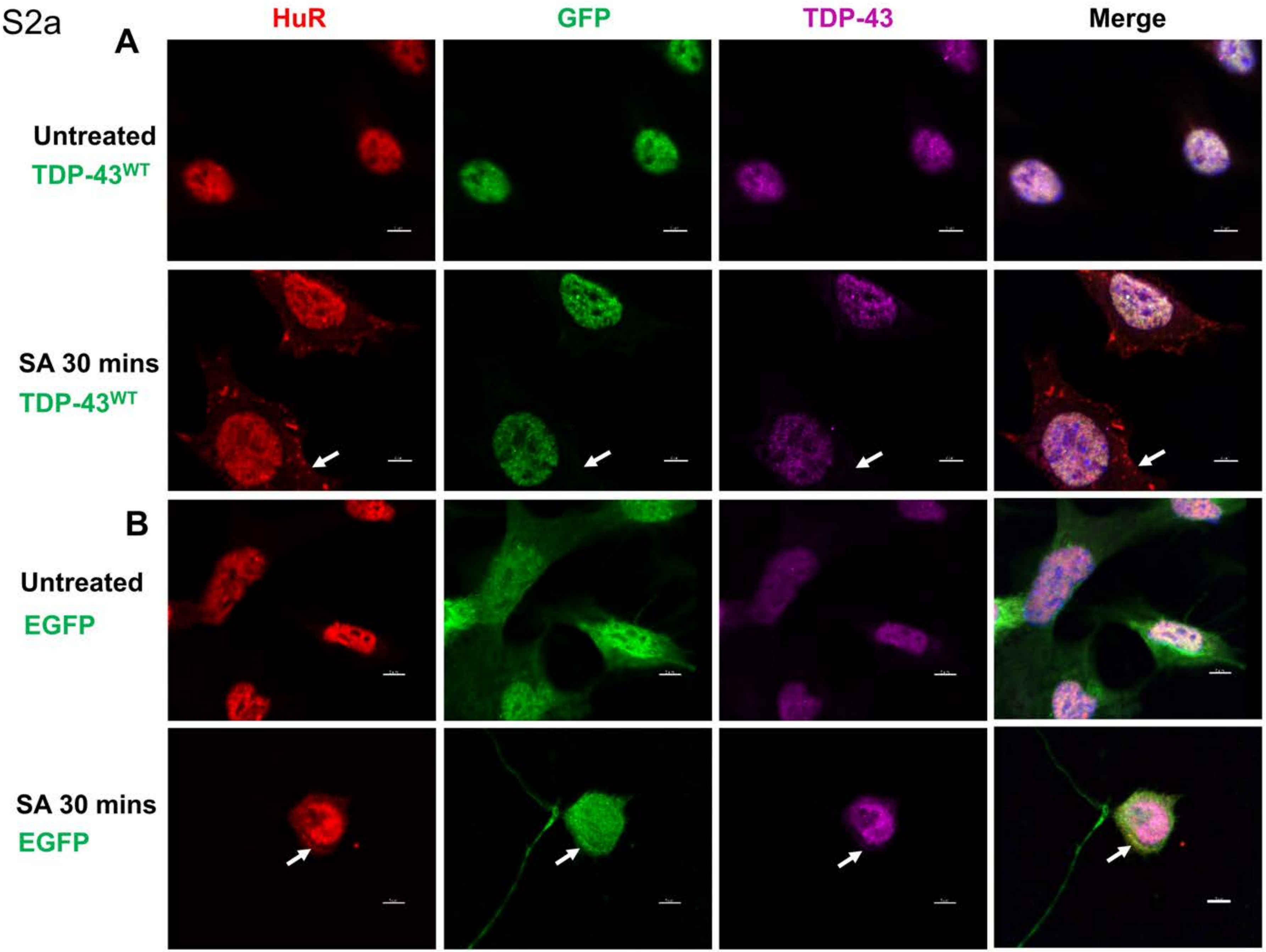
A
HuR
GFP
TDP-43
Merge
Untreated
TDP-43WT
SA 30 mins
TDP-43WT
B
Untreated
EGFP
SA 30 mins
EGFP

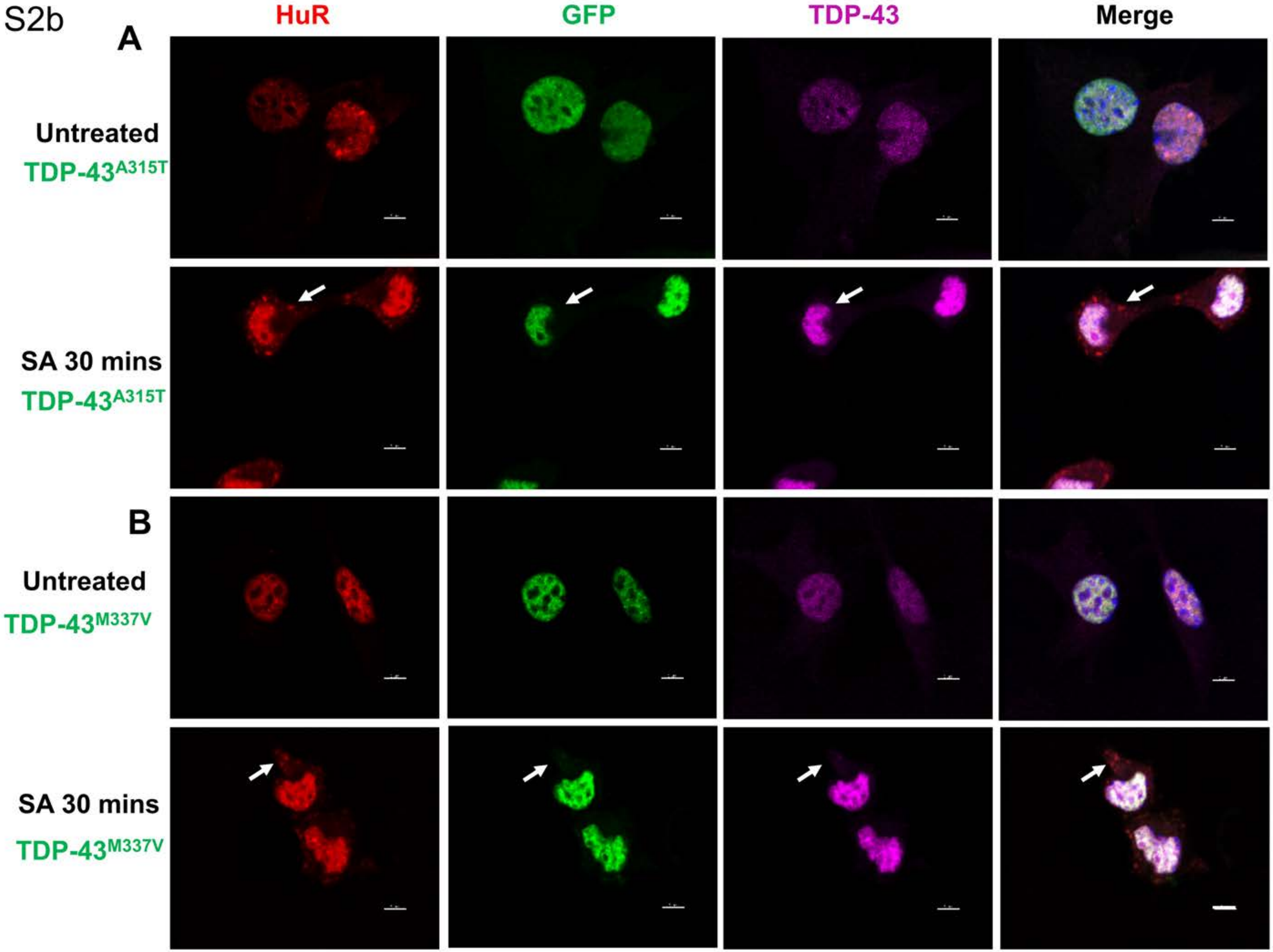

S2b
HuR
GFP
TDP-43
Merge
A
Untreated
TDP-43A315T
SA 30 mins
TDP-43A315T
B
Untreated
TDP-43M337V
SA 30 mins
TDP-43M337V

S3a

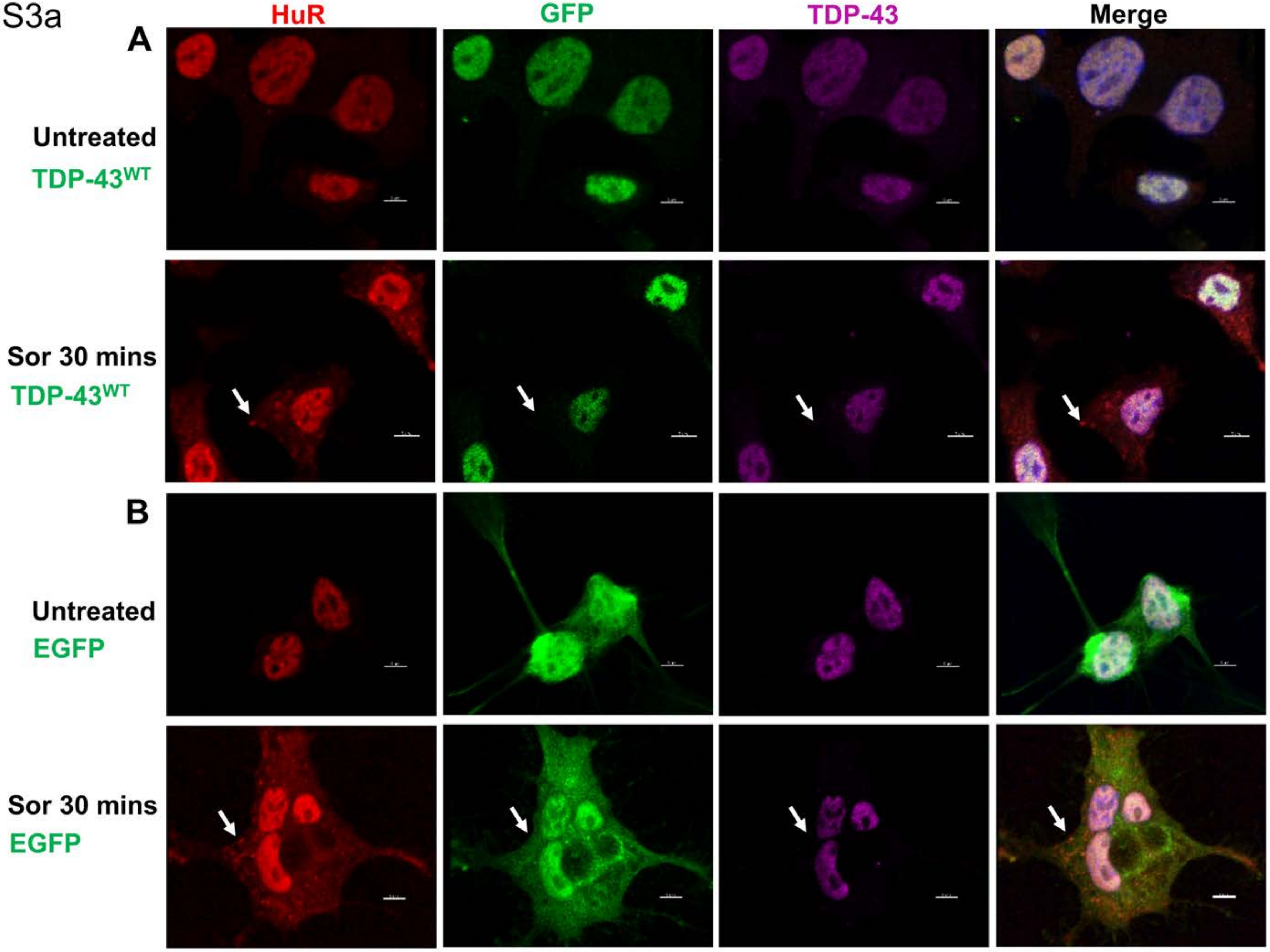
HuR
GFP
TDP-43
Merge
A
Untreated
TDP-43WT
Sor 30 mins
TDP-43WT
B
Untreated
EGFP
Sor 30 mins
EGFP

S3b

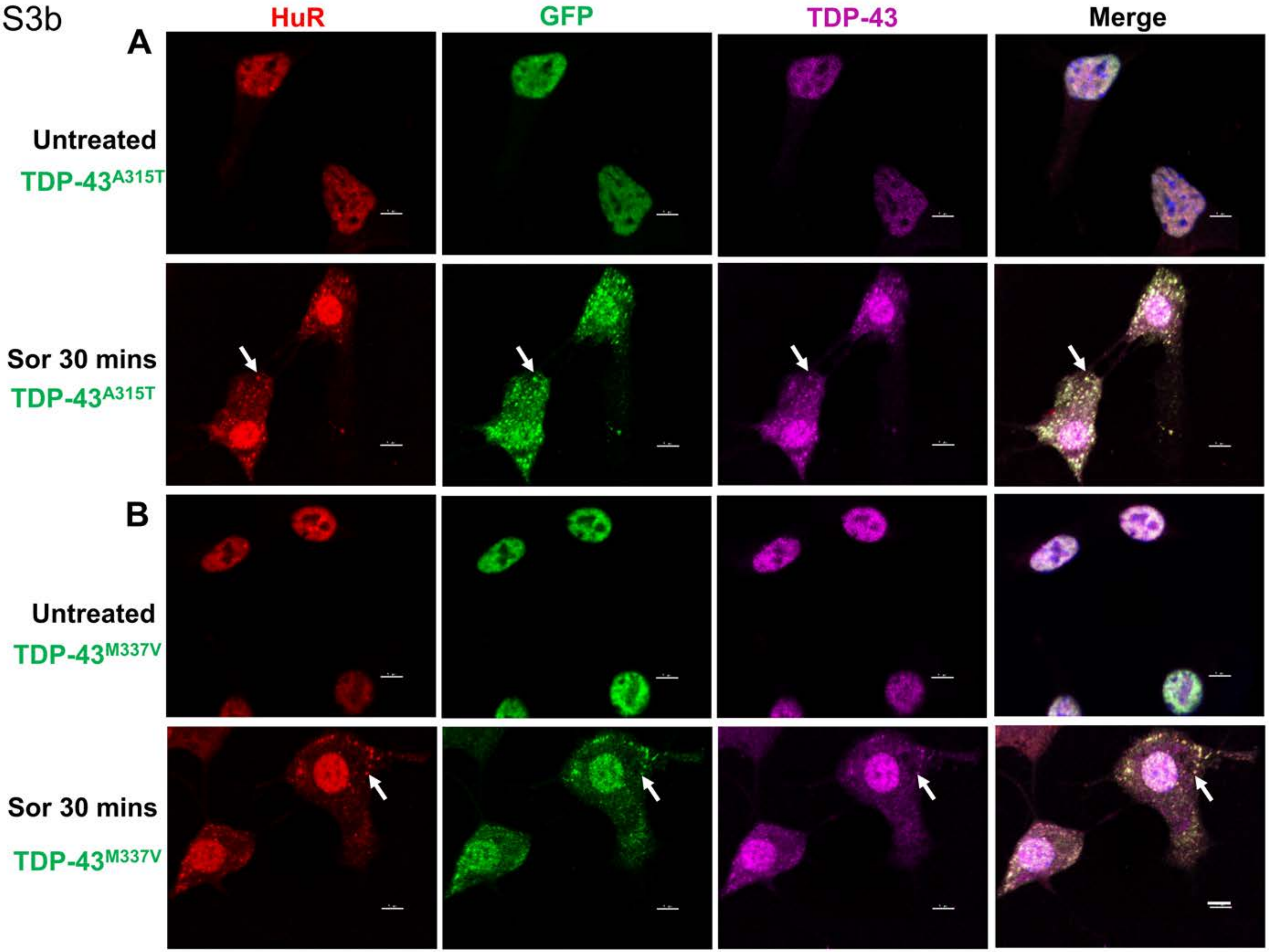
HuR
GFP
TDP-43
Merge
A
Untreated
TDP-43A315T
Sor 30 mins
TDP-43A315T
B
Untreated
TDP-43M337V
Sor 30 mins
TDP-43M337V

S4

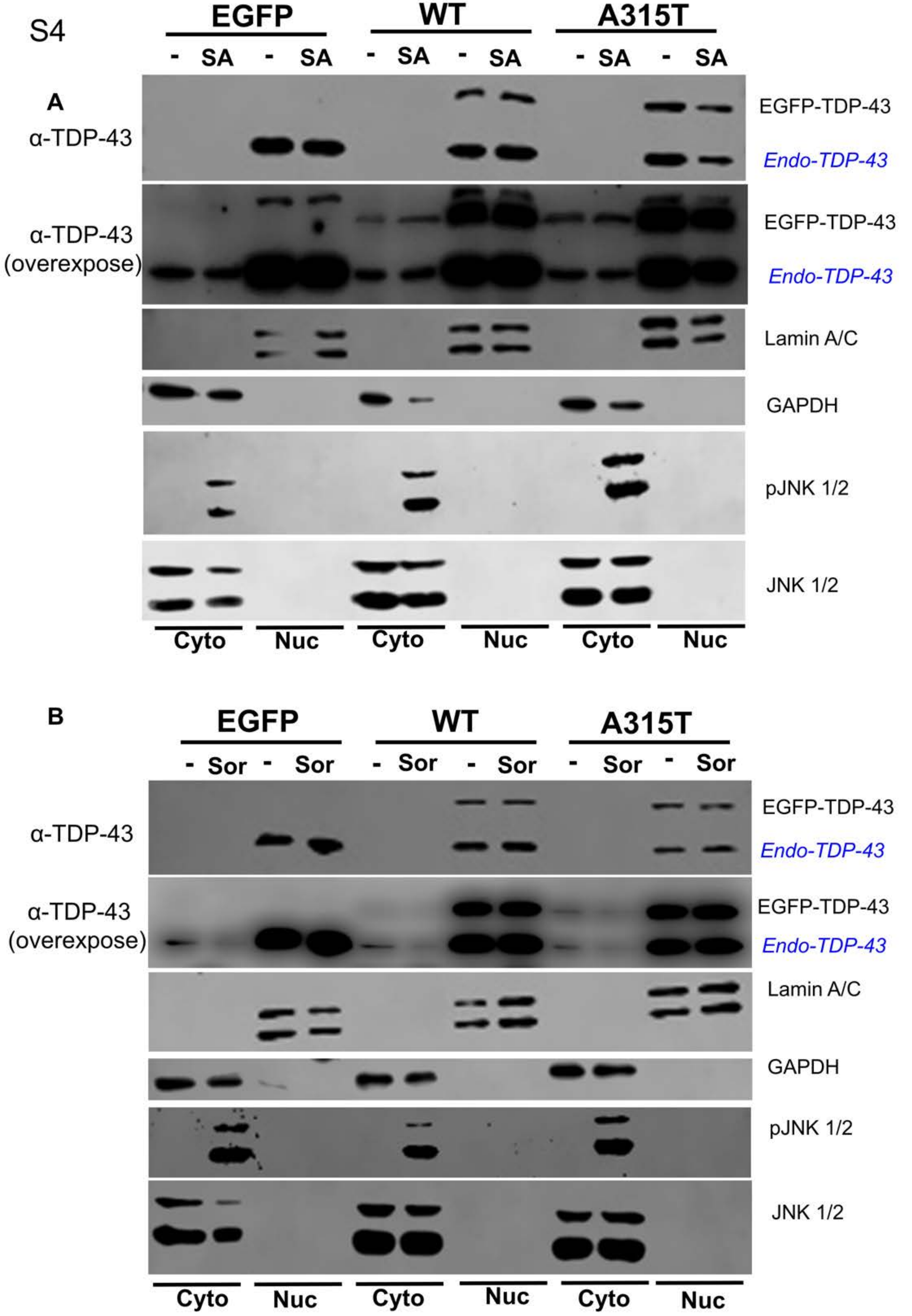

A
EGFP
WT
A315T
- SA - SA - SA - SA - SA - SA
α-TDP-43
α-TDP-43 (overexpose)
EGFP-TDP-43
Endo-TDP-43
Lamin A/C
GAPDH
pJNK 1/2
JNK 1/2
Cyto Nuc Cyto Nuc Cyto Nuc
B
EGFP
WT
A315T
- Sor - Sor - Sor - Sor - Sor - Sor
α-TDP-43
α-TDP-43 (overexpose)
EGFP-TDP-43
Endo-TDP-43
Lamin A/C
GAPDH
pJNK 1/2
JNK 1/2
Cyto Nuc Cyto Nuc Cyto Nuc

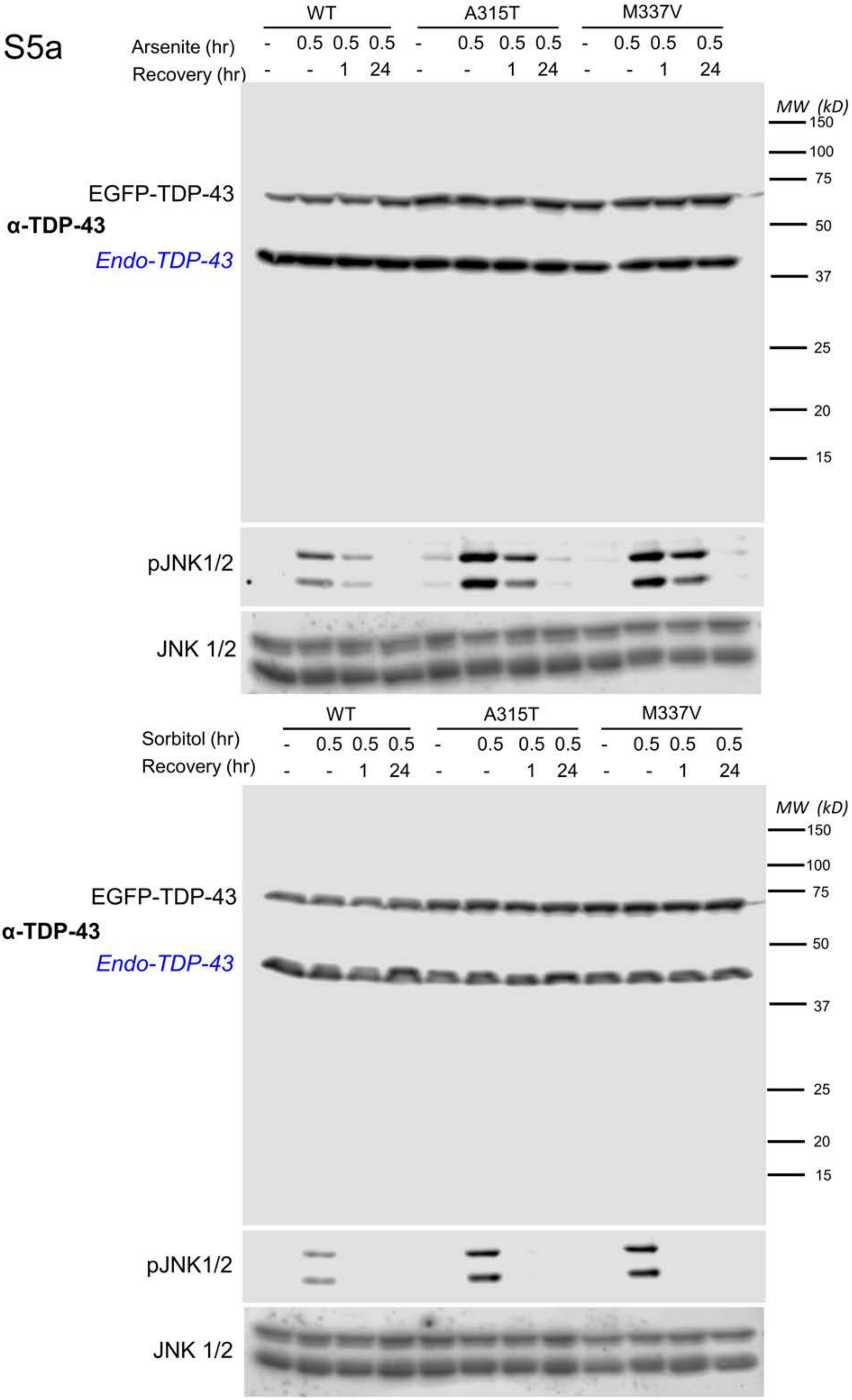

S5a
WT
A315T
M337V
Arsenite (hr) - 0.5 0.5 0.5 - 0.5 0.5 0.5 - 0.5 0.5 0.5
Recovery (hr) - - 1 24 - - 1 24 - - 1 24
MW (kD)
150
100
75
50
37
25
20
15
EGFP-TDP-43
α-TDP-43
Endo-TDP-43
pJNK1/2
JNK 1/2
WT
A315T
M337V
Sorbitol (hr) - 0.5 0.5 0.5 - 0.5 0.5 0.5 - 0.5 0.5 0.5
Recovery (hr) - - 1 24 - - 1 24 - - 1 24
MW (kD)
150
100
75
50
37
25
20
15
EGFP-TDP-43
α-TDP-43
Endo-TDP-43
pJNK1/2
JNK 1/2

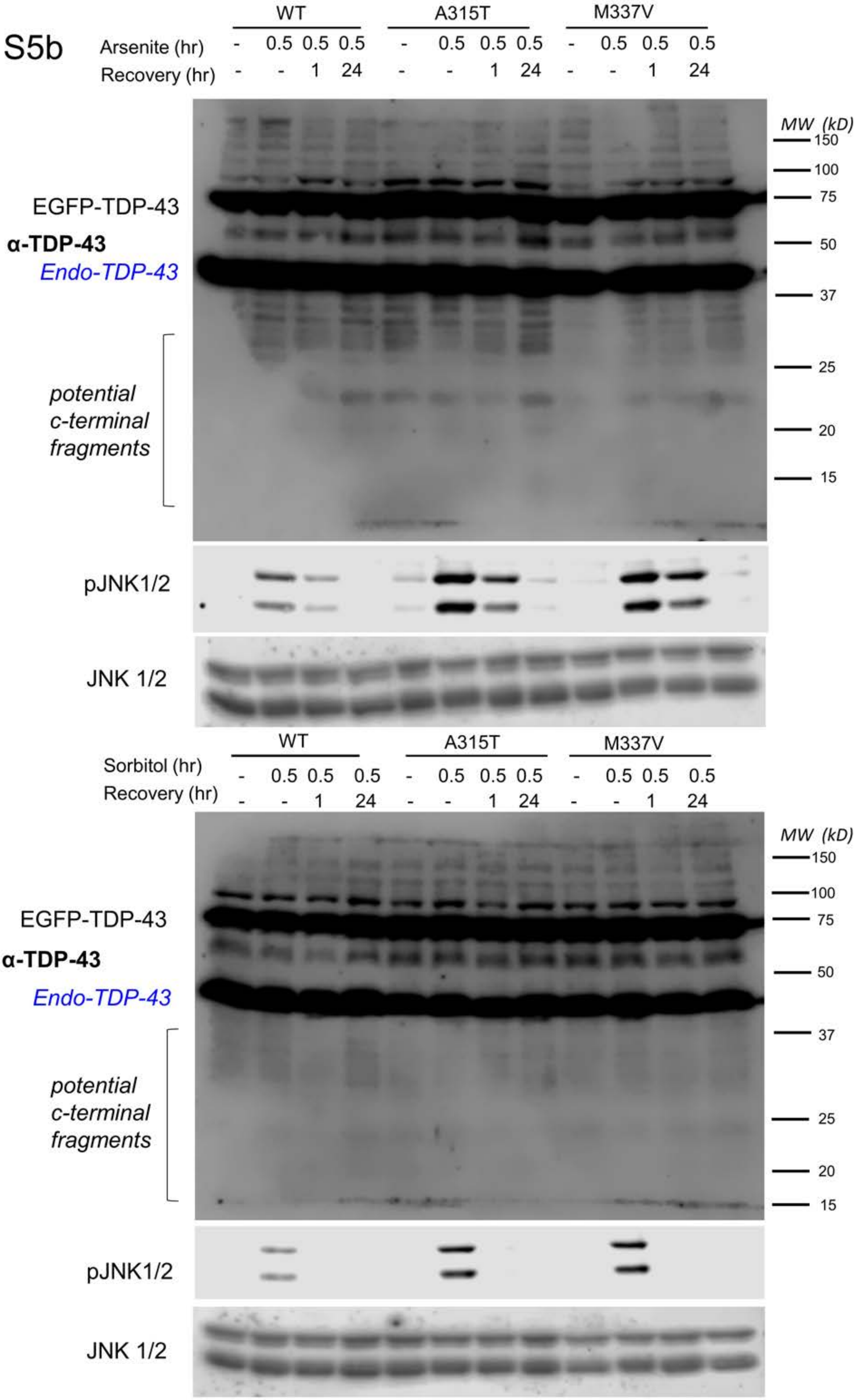
S5b
WT
A315T
M337V
Arsenite (hr) - 0.5 0.5 0.5 - 0.5 0.5 0.5 - 0.5 0.5 0.5
Recovery (hr) - - 1 24 - - 1 24 - - 1 24
MW (kD)
150
100
75
50
37
25
20
15
EGFP-TDP-43
α-TDP-43
Endo-TDP-43
potential c-terminal fragments
pJNK1/2
JNK 1/2
WT
A315T
M337V
Sorbitol (hr) - 0.5 0.5 0.5 - 0.5 0.5 0.5 - 0.5 0.5 0.5
Recovery (hr) - - 1 24 - - 1 24 - - 1 24
MW (kD)
150
100
75
50
37
25
20
15
EGFP-TDP-43
α-TDP-43
Endo-TDP-43
potential c-terminal fragments
pJNK1/2
JNK 1/2

S6

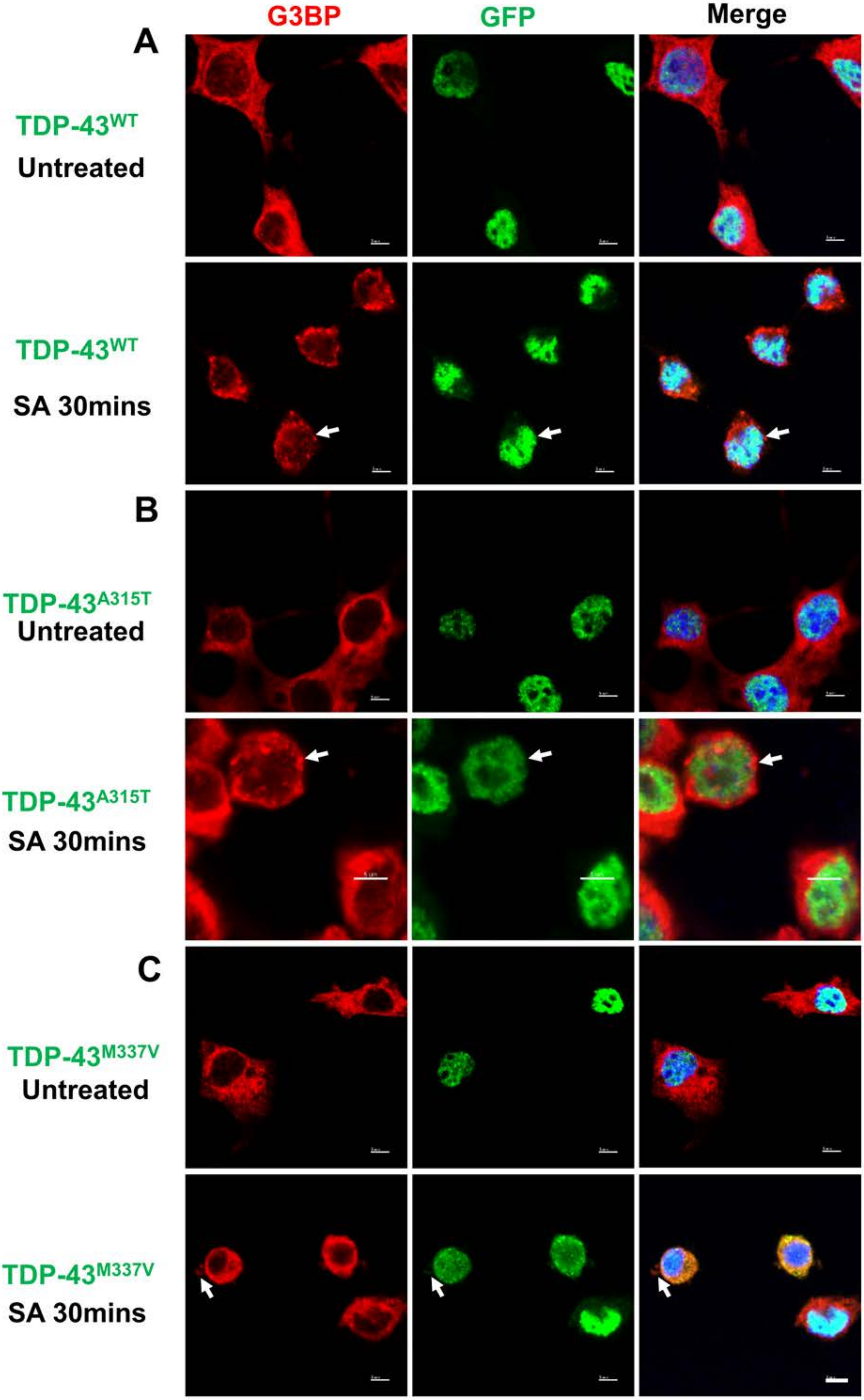
G3BP
GFP
Merge
A
TDP-43WT
Untreated
TDP-43WT
SA 30mins
B
TDP-43A315T
Untreated
TDP-43A315T
SA 30mins
C
TDP-43M337V
Untreated
TDP-43M337V
SA 30mins

S7

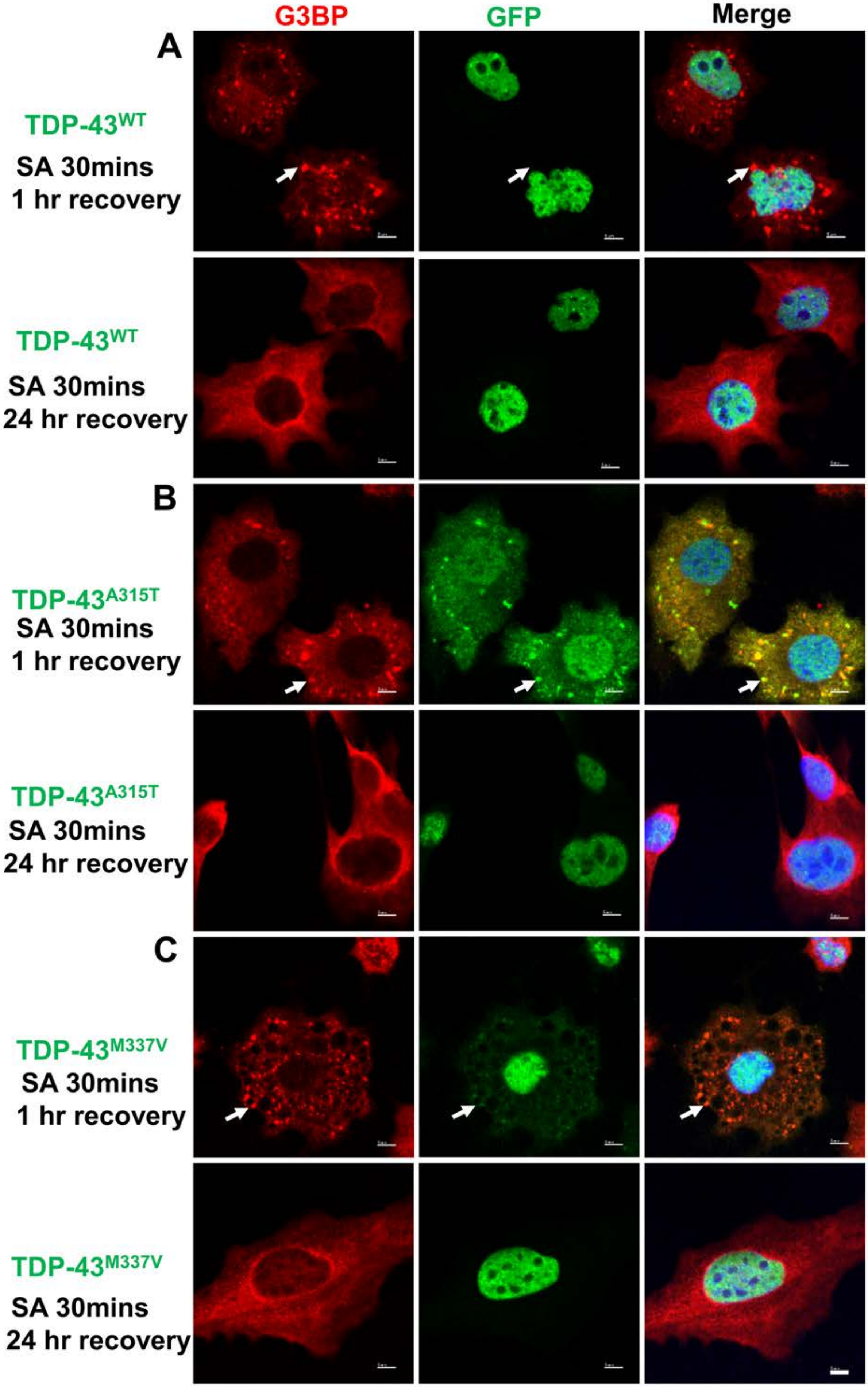
G3BP
GFP
Merge
A
TDP-43WT
SA 30mins
1 hr recovery
TDP-43WT
SA 30mins
24 hr recovery
B
TDP-43A315T
SA 30mins
1 hr recovery
TDP-43A315T
SA 30mins
24 hr recovery
C
TDP-43M337V
SA 30mins
1 hr recovery
TDP-43M337V
SA 30mins
24 hr recovery

S8

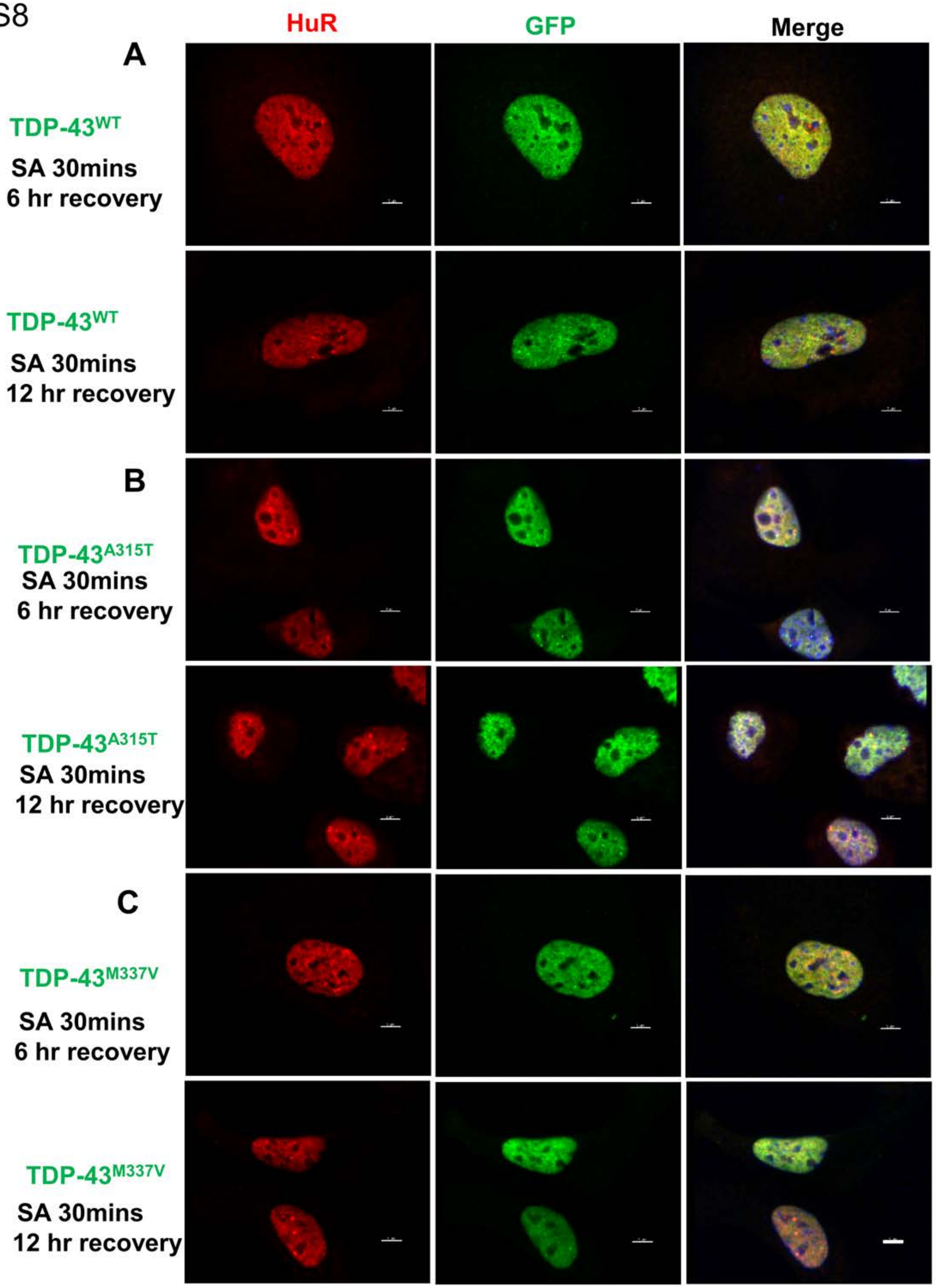
HuR
GFP
Merge
A
TDP-43WT
SA 30mins
6 hr recovery
TDP-43WT
SA 30mins
12 hr recovery
B
TDP-43A315T
SA 30mins
6 hr recovery
TDP-43A315T
SA 30mins
12 hr recovery
C
TDP-43M337V
SA 30mins
6 hr recovery
TDP-43M337V
SA 30mins
12 hr recovery

S9

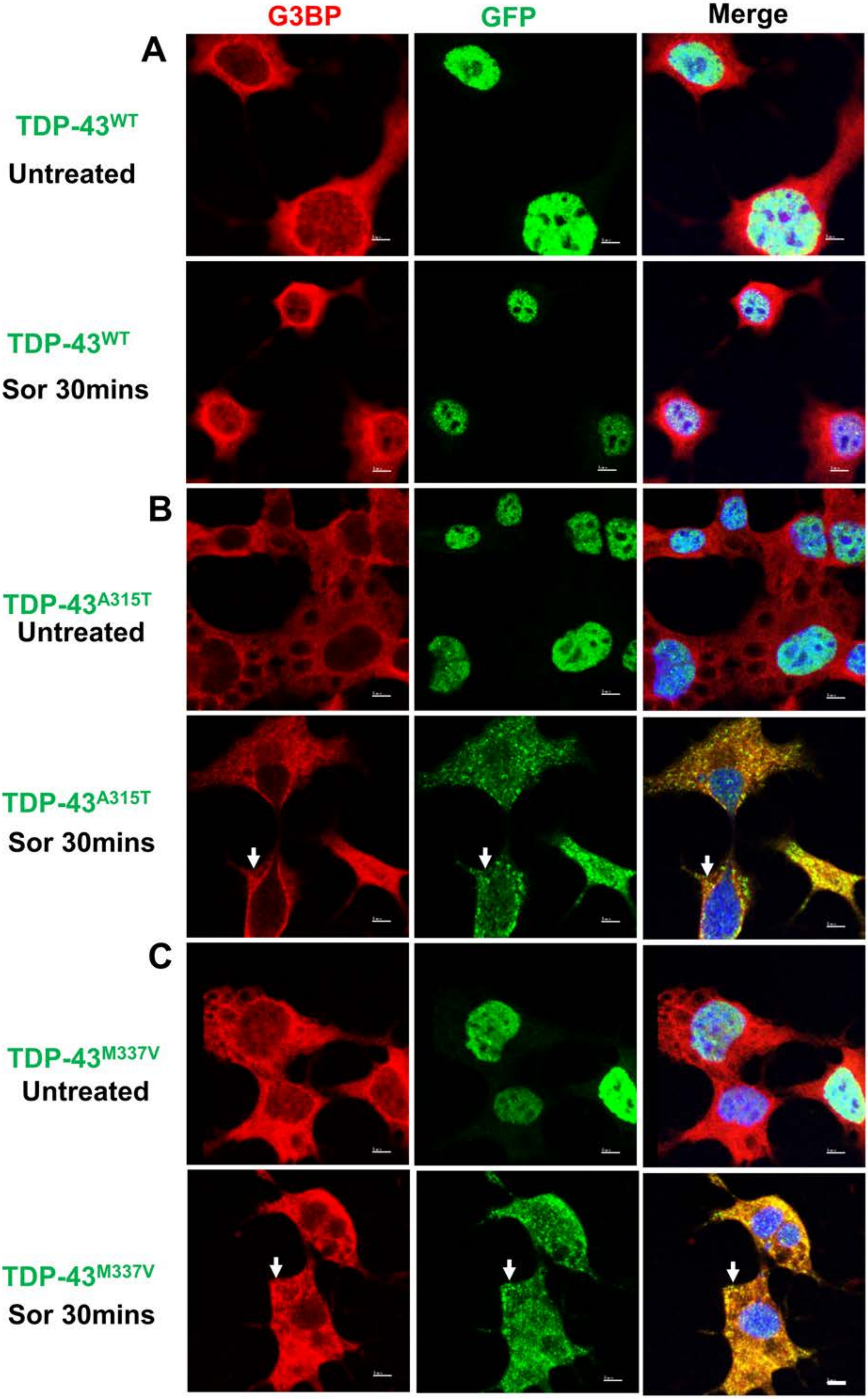
G3BP
GFP
Merge
A
TDP-43WT
Untreated
TDP-43WT
Sor 30mins
B
TDP-43A315T
Untreated
TDP-43A315T
Sor 30mins
C
TDP-43M337V
Untreated
TDP-43M337V
Sor 30mins

S10

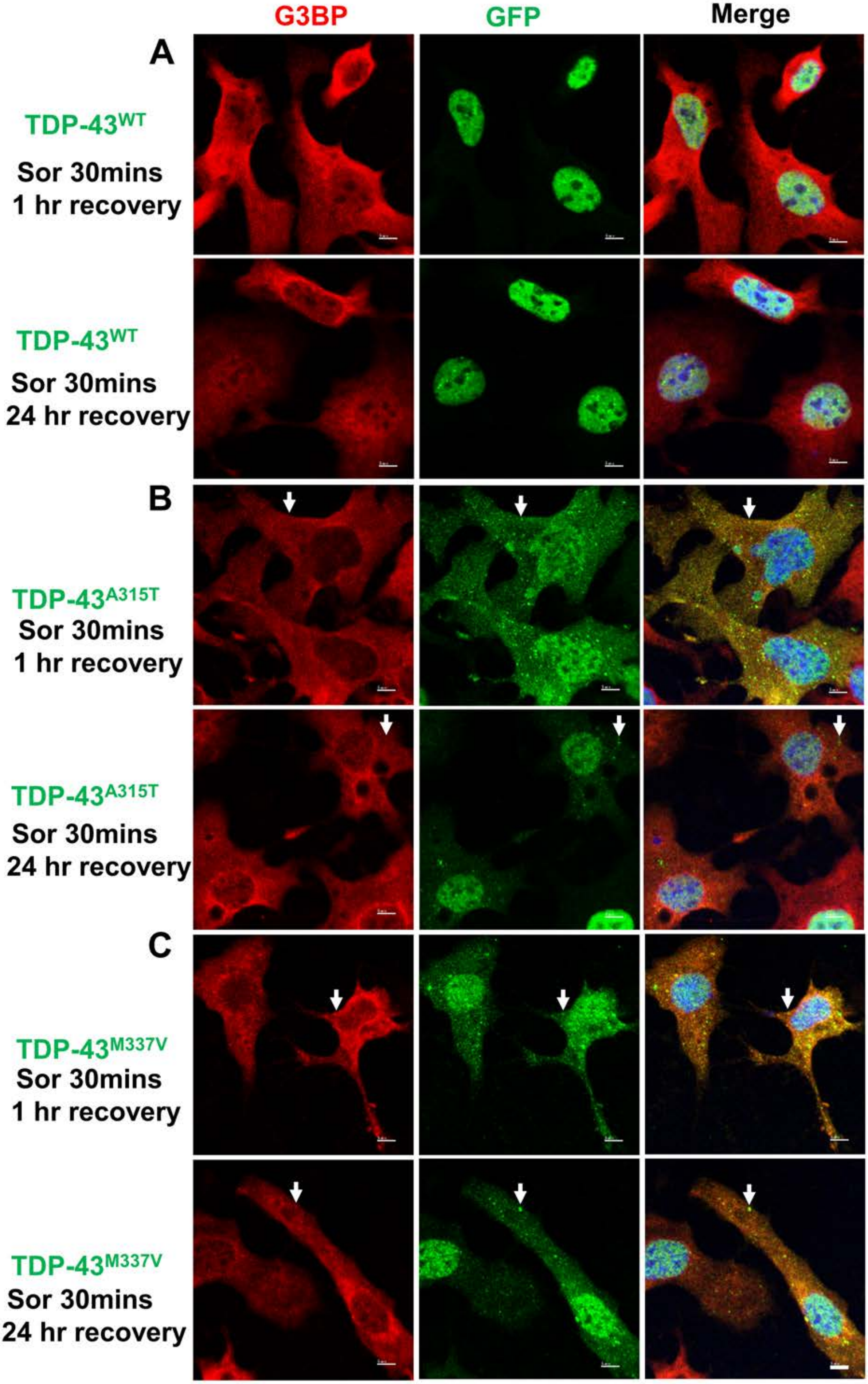
G3BP
GFP
Merge
A
TDP-43WT
Sor 30mins
1 hr recovery
TDP-43WT
Sor 30mins
24 hr recovery
B
TDP-43A315T
Sor 30mins
1 hr recovery
TDP-43A315T
Sor 30mins
24 hr recovery
C
TDP-43M337V
Sor 30mins
1 hr recovery
TDP-43M337V
Sor 30mins
24 hr recovery
